# Soluble IL-33 receptor sST2 inhibits colorectal cancer malignant growth by modifying the tumour microenvironment

**DOI:** 10.1038/ncomms13589

**Published:** 2016-11-24

**Authors:** Miho Akimoto, Riruke Maruyama, Hiroyuki Takamaru, Takahiro Ochiya, Keizo Takenaga

**Affiliations:** 1 Department of Life Science, Shimane University Faculty of Medicine, 89-1 Enya, Izumo, Shimane 693-8501, Japan; 2 Department of Pathology, Shimane University Faculty of Medicine, Izumo, Shimane 693-8501, Japan; 3 Endoscopy Division, National Cancer Center Hospital, Tokyo 104-0045, Japan; 4 Division of Molecular and Cellular Medicine, National Cancer Center Research Institute, Tokyo 104-0045, Japan

## Abstract

Interleukin-33 (IL-33) was recently shown to be involved in the inflammatory tumour microenvironment and the progression of colorectal cancer (CRC). We report here that the expression level of sST2, a soluble form of the IL-33 receptor (ST2L), is inversely associated with the malignant growth of CRC. sST2 is downregulated in high-metastatic cells compared with low-metastatic human and mouse CRC cells. Knockdown of sST2 in low-metastatic cells enhances tumour growth, metastasis and tumour angiogenesis, whereas its overexpression in high-metastatic cells suppresses these processes. Circulating and intratumourally administered sST2-Fc fusion protein reduce tumour growth, metastatic spread and tumour angiogenesis in mice bearing high-metastatic CRC. Mechanistically, sST2 suppresses IL-33-induced angiogenesis, Th1- and Th2-responses, macrophage infiltration and macrophage M2a polarization. In conclusion, we show that sST2 negatively regulates tumour growth and the metastatic spread of CRC through modification of the tumour microenvironment. Thus, the IL-33/ST2L axis may be a potential therapeutic target in CRC.

## Abstract (web summary)

IL-33 is a pro-inflammatory cytokine with a role in colorectal cancer. Here, the authors show that circulating tumour-derived sST2, an IL-33 decoy receptor, delayed the growth and progression of colorectal cancer cells by inhibiting Th1/Th2 polarization, macrophage infiltration and angiogenesis.

## Graphical abstract

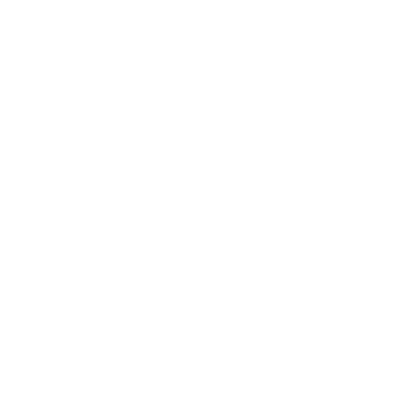

Colorectal cancer (CRC) is the fourth-leading cause of cancer death in the world, with 50,000 patients dying each year as a result of metastatic disease that is refractory to systemic therapy^1^,^2^. Because CRC progression is closely related to inflammation, a better understanding of the inflammatory tumour microenvironment of CRC is critical for the development of more effective therapeutic approaches for patients with metastatic CRC.

ST2 is a member of the interleukin-1 receptor family that was originally identified as a responsive gene in serum- or oncogene-stimulated mouse fibroblasts^3^. *ST2* pre-mRNA produces at least three isoforms through alternative splicing: sST2 (a soluble form), ST2L (a transmembrane form) and ST2V (a variant ST2)^4^,^5^,^6^. ST2L is expressed on the membrane of a variety of cell types, including Th2 lymphocytes, macrophages, mast cells, basophils, eosinophils, dendritic cells, and NK and iNKT cells^7^,^8^,^9^, whereas sST2 is predominantly expressed on fibroblasts and epithelial cells^10^,^11^.

Interleukin-33 (IL-33) is a member of the interleukin-1 (IL-1) family and has been identified as a natural ligand for ST2L^12^,^13^. IL-33 has been shown to be primarily expressed as a pro-inflammatory cytokine by epithelial cells, myofibroblasts, fibroblasts, adipocytes, endothelial cells, smooth muscle cells and macrophages, either constitutively or in response to different stimuli^14^,^15^,^16^. IL-33 binds to the heterodimeric complex consisting of ST2L and IL-1 receptor accessory protein (IL-1RAP)^13^. sST2 interferes with this interaction as a decoy receptor^10^,^11^. The IL-33/ST2L axis induces the production of both pro- and anti-inflammatory cytokines through the recruitment of myeloid differentiation primary response 88 (MyD88) and subsequent activation of NF-κB signalling^13^. Excessive stimulation of the IL-33/ST2L axis promotes rheumatic and airway inflammatory diseases, anaphylactic shock, and inflammatory and fibrotic disorders of the gastrointestinal tract^5^,^6^,^15^,^16^,^17^.

Recent studies have shown a relationship between the IL-33/ST2L axis and the progression of cancer. IL-33 serum levels were positively correlated with a poor prognosis in gastric cancer^18^, non-small-cell lung cancer^19^ and hepatocellular carcinoma^20^. The IL-33/ST2L axis promoted tumour progression in a breast cancer mouse model by diminishing innate anti-tumour immunity resulting from the accumulation of immunosuppressive cells^21^. Recently, a link was reported between inflammation (more specifically the IL-33/ST2L axis) and CRC progression, including the promotion of intestinal polyposis, the progression of colorectal adenoma to carcinoma and the enhancement of metastasis^22^,^23^,^24^,^25^. We have also reported that IL-33 promotes 3LL lung cancer progression by selecting for more ST2L-negative metastatic cells in the tumour microenvironment^26^. Conversely, IL-33 attenuated tumour growth and metastasis in the B16 melanoma, 3LL and 4T1 mammary tumour models by increasing the cytotoxicity and tumour infiltration of CD8^+^ T cells and NK cells^27^,^28^. Thus, although the role of the IL-33/ST2L axis in regulating tumour progression is controversial, understanding its regulation may provide us with valuable information for controlling the malignant behaviour of CRC.

In this study, we focused on sST2 expression in human and mouse CRC cells and report that sST2 level is inversely correlated with their malignant growth *in vivo*. Importantly, we demonstrate the suppressive effect of circulating sST2 and sST2-Fc fusion protein on the tumour growth and distant metastasis of highly metastatic CRC.

## Results

### ST2 expression in human CRC samples

**Figure 1:**
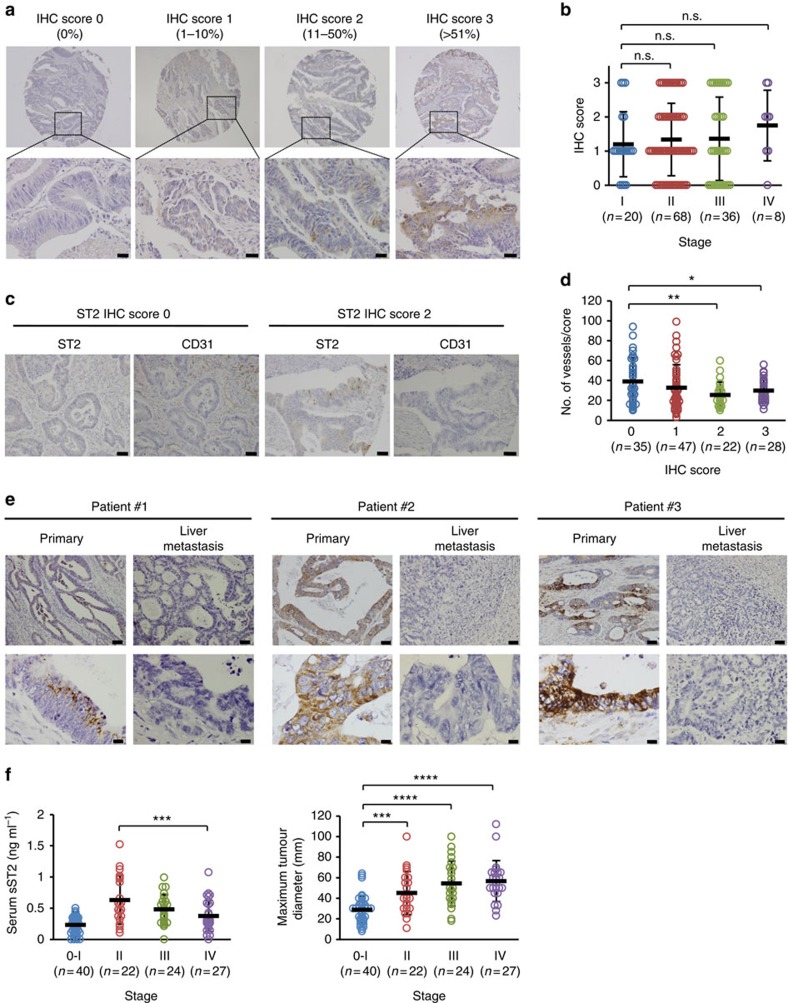
ST2 expression in human CRC samples. (**a**) IHC analysis of ST2 expression using human primary CRC tissue arrays. The IHC score was expressed based on the percentage of ST2-positive cells within the total cancerous cells as follows: IHC score 1, 0% positive; IHC score 2, 1–10% positive; IHC score 3, 11–50% positive; IHC score, >50% positive. Scale bars: 100 μm. (**b**) Correlation between CRC stages and ST2 IHC scores. (**c**) IHC analysis of ST2 and CD31 expression in consecutive tissue cores. Scale bars: 100 μm. (**d**) Correlation between CRC stages and the number of tumour microvessels. (**e**) IHC analysis of ST2 expression in matched primary tumour and liver metastatic lesion samples from CRC patients. Scale bars: 100 and 20 μm for the upper and lower panels, respectively. Note that the ST2 immunostaining was localized in the cytoplasm but not on the cell membranes of the cancerous cells from patients #1 and #2. (**f**) Serum sST2 levels of patients with various stages of CRC. Left: sST2 level in the sera from patients with various stages of CRC. Right: Maximum tumour diameter of CRC in patients in various CRC stages. The data are shown as the mean±s.d. n.s. not significant, *P<0.05, **P<0.02, ***P<0.001, ****P<0.0001 by Student's *t*-test.

To investigate the relationship between ST2 expression and malignant growth in human CRC, we first performed immunohistochemistry (IHC) using primary CRC tissue arrays. For this, we used an antibody that reacts with both ST2L and sST2 due to a lack of an sST2-specific antibody. The results showed that ST2 was heterogeneously expressed in cancerous cells but not in stromal cells (Fig. 1a). We could not find an association between the ST2 IHC score and the tumour stage (Fig. 1b). Nevertheless, interestingly, an association between a low ST2 IHC score and a greater microvessel density emerged by using duplicate tissue cores for each case (Fig. 1c,d). Furthermore, IHC of matched primary lesions and liver metastatic lesion samples from CRC patients revealed that ST2 expression was decreased in the metastatic lesions compared to the primary lesions in 9 of 16 patients. Among them, the liver metastatic foci of three patients were completely negative for ST2 immunostaining, whereas the matched primary lesion was positive (Fig. 1e). Notably, ST2 immunoreactivity was localized in the cytoplasm as dots but not on the plasma membrane of CRC cells from patients #1 and #2 (Fig. 1e), suggesting that primary CRC cells of the patients preferentially produce secretory sST2. To obtain more compelling evidence, we compared the serum sST2 level among patients in various stages of CRC. Intriguingly, low serum sST2 levels tended to be associated with more advanced tumour stages (Stage II versus Stage IV, *P*=0.008), and the maximum tumour diameter of the patients was almost comparable (Fig. 1f). Although patients with Stage 0-I CRC had lower serum sST2 levels than patients with other stages of CRC, this may be attributed to smaller tumour sizes (Fig. 1f) and a number of intramucosal cancers from which sST2 may barely be released into the bloodstream. Altogether, these results implied that low sST2 expression was linked to more malignant growth in CRC patients.

### sST2 inhibits the malignant growth of human CRC cells

**Figure 2:**
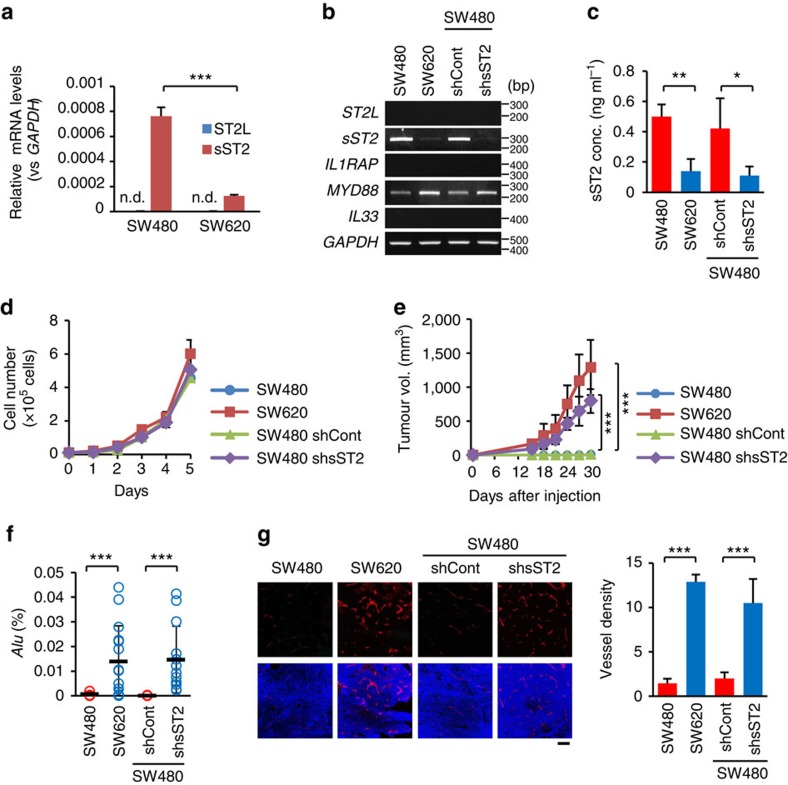
sST2 inhibits the malignant growth of human CRC cells. (**a**) qRT-PCR analysis of *ST2L* and *sST2* mRNA expression in SW480 and SW620 cell lines. The expression levels are presented as the ratio to the expression of *GAPDH* (*n*=3). (**b**) RT-PCR analysis of *ST2L*, *sST2*, *IL1RAP*, *MyD88* and *IL33* mRNA expression in SW480, SW620, SW480-shCont and SW480-shsST2 cells. *GAPDH* served as the loading control. (**c**) Secretion of sST2 in the culture supernatants by SW480, SW620, SW480-shCont and SW480-shsST2 cells (*n*=3). (**d**) *In vitro* growth of SW480, SW620, SW480-shCont and SW480-shsST2 cells (*n*=3). (**e**) Tumour growth of SW480, SW620, SW480-shCont and SW480-shsST2 cells. The cells (1 × 10^6^ cells) were subcutaneously implanted with 50% Matrigel into BALB/c nude mice (*n*=7 mice per group). (**f**) qPCR analysis of *Alu* sequence in the lungs of mice bearing SW480 (three different portions excised from a tumour), SW620 (15 different portions excised from 7 lungs), SW480-shCont (three different portions excised from a lung) and SW480-shsST2 tumours (13 different portions excised from 7 lungs). (**g**) Tumour angiogenesis. CD31 staining of cryostat sections of tumours formed by SW480, SW620, SW480-shCont and SW480-shsST2 cells (left) and the vessel density in the tumours (right) (*n*=15 fields). Scale bar: 200 μm. The data are shown as the mean±s.d. n.d. not detected. *P<0.05, **P<0.01, ***P<0.001 by Student's *t*-test.

In accord with these results, sST2 expression was significantly higher in human CRC SW480 cells than in isogenic SW620 cells, which were derived from the primary and secondary tumours from a single patient, at both the mRNA and protein levels (Fig. 2a–c). Neither of these cell lines expressed *ST2L* or *IL1RAP* mRNA (Fig. 2b), indicating that both of cell lines were unresponsive to exogenous IL-33. Thus, these data support the hypothesis that sST2 might act as a negative regulator of CRC cell malignancy. To test this hypothesis, we knocked down sST2 expression in SW480 cells with *sST2* short hairpin RNA (shRNA) (Fig. 2b,c) and examined their growth in nude mice compared to parental SW480 and SW620 cells. The control shRNA-expressing cells (SW480-shCont) and *sST2* shRNA-expressing cells (SW480-shsST2) showed comparable growth rates *in vitro* (Fig. 2d). Interestingly, the SW480-shsST2 cells efficiently formed tumours (7 out of 7 mice), whereas the SW480-shCont cells seldom formed tumours (1 out of 7 mice) (Fig. 2e). Furthermore, although we could not observe visible metastatic nodules in the lungs, *Alu* PCR analysis demonstrated the presence of more metastatic cells in the lungs of nude mice bearing SW480-shsST2 and SW620 tumours than those bearing SW480-shCont and SW480 tumours (Fig. 2f). Notably, CD31 staining revealed a greater tumour vessel density in SW480-shsST2 tumours than in SW480-shCont tumours, which were nearly comparable to the tumour vessel density observed in SW620 tumours (Fig. 2g). These results suggest that sST2 inhibits the tumour growth and metastatic spread of human CRC cells, probably through retardation of tumour angiogenesis.

### sST2 inhibits the malignant growth of mouse CRC cells

To extend the above findings, we examined the effect of sST2 expression on the malignant growth of mouse CRC cell lines (low-metastatic NM11 cells and high-metastatic LuM1 cells). NM11 cells expressed high levels of *sST2* mRNA, whereas LuM1 cells expressed small amounts (Supplementary Fig. 1a). NM11 cells also expressed *ST2L* and *Il1rap* mRNAs at higher levels than LuM1 cells (Supplementary Fig. 1a). The LuM1 cells grew slightly faster than the NM11 cells under normal culture conditions (Supplementary Fig. 1b), invaded more readily through Matrigel in the presence of transforming growth factor (TGF)-β (a well-known inducer of the epithelial-mesenchymal transition) (Supplementary Fig. 1c), and developed significantly larger tumours and greater numbers of spontaneous pulmonary metastatic nodules than the NM11 cells (Supplementary Fig. 1d,e) following subcutaneous implantation into immunocompetent mice. Moreover, the tumour vessel density was significantly higher in LuM1 than in NM11 tumours (Supplementary Fig. 1f).

**Figure 3:**
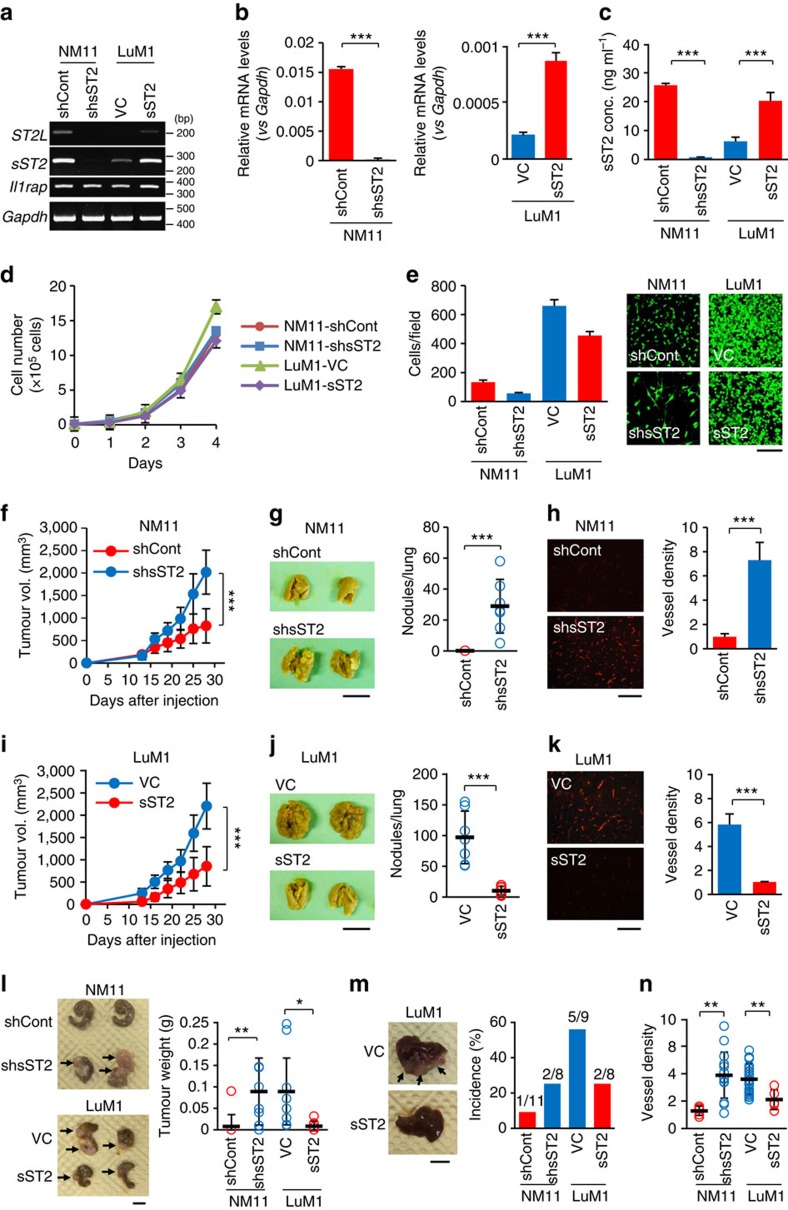
sST2 inhibits the malignant growth of mouse CRC cells. (**a**) RT-PCR analysis of *ST2L*, *sST2* and *Il1rap* mRNA expression in NM11-shCont, NM11-shsST2, LuM1-VC and LuM1-sST2 cells. *Gapdh* served as the loading control. (**b**) qRT-PCR analysis of *ST2L* and *sST2* mRNA expression in NM11-shCont, NM11-shsST2, LuM1-VC and LuM1-sST2 cells. The expression levels are presented as the ratio to the expression of *Gapdh* mRNA (*n*=3). (**c**) Secretion of sST2 into the culture supernatants by NM11-shCont, NM11-shsST2, LuM1-VC and LuM1-sST2 cells (*n*=3). (**d**) *In vitro* growth of NM11-shCont, NM11-shsST2, LuM1-VC and LuM1-sST2 cells (*n*=3). (**e**) Invasive ability of NM11-shCont, NM11-shsST2, LuM1-VC and LuM1-sST2 cells (*n*=15 fields). Scale bar: 200 μm. (**f**) Tumour growth formed by NM11-shCont and NM11-shsST2 cells. The cells (3 × 10^5^ cells) were subcutaneously implanted with 50% Matrigel into BALB/c mice (*n*=7 mice per group). (**g**) Spontaneous lung metastasis of the tumours formed by NM11-shCont and NM11-shsST2 cells (*n*=7 mice per group). Scale bar: 1 cm. (**h**) Tumour angiogenesis. CD31 staining of cryostat sections of the tumours formed by NM11-shCont and NM11-shsST2 cells (left) and the vessel density (right) (*n*=15 fields). Scale bar: 100 μm. (**i**) Tumour growth formed by LuM1-VC and LuM1-sST2 cells. The cells (3 × 10^5^ cells) were subcutaneously implanted with 50% Matrigel into BALB/c mice (*n*=7 mice per group). (**j**) Spontaneous lung metastasis of the tumours formed by LuM1-VC and LuM1-sST2 cells (*n*=7 mice per group). Scale bar: 1 cm. (**k**) Tumour angiogenesis. CD31 staining of cryostat sections of the tumours formed by LuM1-VC and LuM1-sST2 cells (left) and the vessel density (right) (*n*=15 fields). Scale bar: 100 μm. (**l**) Orthotopic tumour growth. NM11-shCont, NM11-shsST2, LuM1-VC and LuM1-sST2 cells (1.5 × 10^5^ cells) were implanted into the caecal walls of BALB/c mice (*n*=11, 8, 9 and 8 mice, respectively). Scale bar: 1 cm. (**m**) Incidence of spontaneous macroscopic liver metastasis of the tumours formed by NM11-shCont, NM11-shsST2, LuM1-VC and LuM1-sST2 cells. Scale bar: 1 cm. (**n**) Vessel density as assessed by CD31 staining. *n*=5, 15, 30 and 5 fields for NM11-shCont, NM11-shsST2, LuM1-VC and LuM1-sST2 tumours, respectively. The data are shown as the mean±s.d. *P<0.02, **P<0.005, ***P<0.001 by Student's *t*-test.

We stably knocked down endogenous sST2 in NM11 cells by transfecting five different lentivirus-encoded shRNAs and established respective cell lines. To examine the inherent role of sST2, we employed one of the cell lines (NM11-shsST2 cells) for subsequent studies because it demonstrated sST2 downregulation at the mRNA and protein levels (Fig. 3a–c). We also established NM11 cells transduced with an empty lentivirus vector as controls (NM11-shCont). Cell proliferation was not affected by sST2 depletion in NM11 cells, and the invasive ability was slightly reduced (Fig. 3d,e). In contrast, tumour growth, spontaneous metastasis and angiogenesis of the NM11-shsST2 subcutaneous tumours were markedly enhanced compared with the NM11-shCont tumours (Fig. 3f–h). Thus, similar to SW480 cells, sST2 knockdown in NM11 cells resulted in the enhancement of malignant growth *in vivo*.

Next, we established stable sST2-overexpressing LuM1 cells (LuM1-sST2) by transfecting an sST2 expression vector (pcDNA3.1-sST2). The LuM1-sST2 cells showed increased sST2 expression of approximately 4.3-fold and 3.2-fold at the mRNA and protein levels, respectively, compared with the empty vector-transfected control cells (LuM1-VC) (Fig. 3a–c). Overexpression of sST2 in LuM1 cells did not affect cell proliferation (Fig. 3d). sST2-overexpessing cells showed a decrease in Matrigel invasion of approximately 30% but still exhibited high invasive potential (Fig. 3e). Remarkably, sST2 overexpression resulted in significant suppression of subcutaneous tumour growth, spontaneous metastasis and tumour angiogenesis (Fig. 3i–k). Similar suppressive effects were also observed following sST2 overexpression using another expression vector (Supplementary Fig. 2).

We next examined whether the above results were reproducible in an orthotopic implantation model. To this end, NM11-shCont, NM11-shsST2, LuM1-VC and LuM1-sST2 cells were injected into the caecal wall. The results showed that NM11-shsST2 and LuM1-VC tumours grew more rapidly, metastasized to the liver more efficiently and had a greater number of tumour vessels than NM11-shCont and LuM1-sST2 tumours (Fig. 3l–n). Together, these data provide evidence that sST2 can inhibit the malignant growth of CRC cells *in vivo*.

### Circulating sST2 inhibits the malignant growth of CRC cells

**Figure 4:**
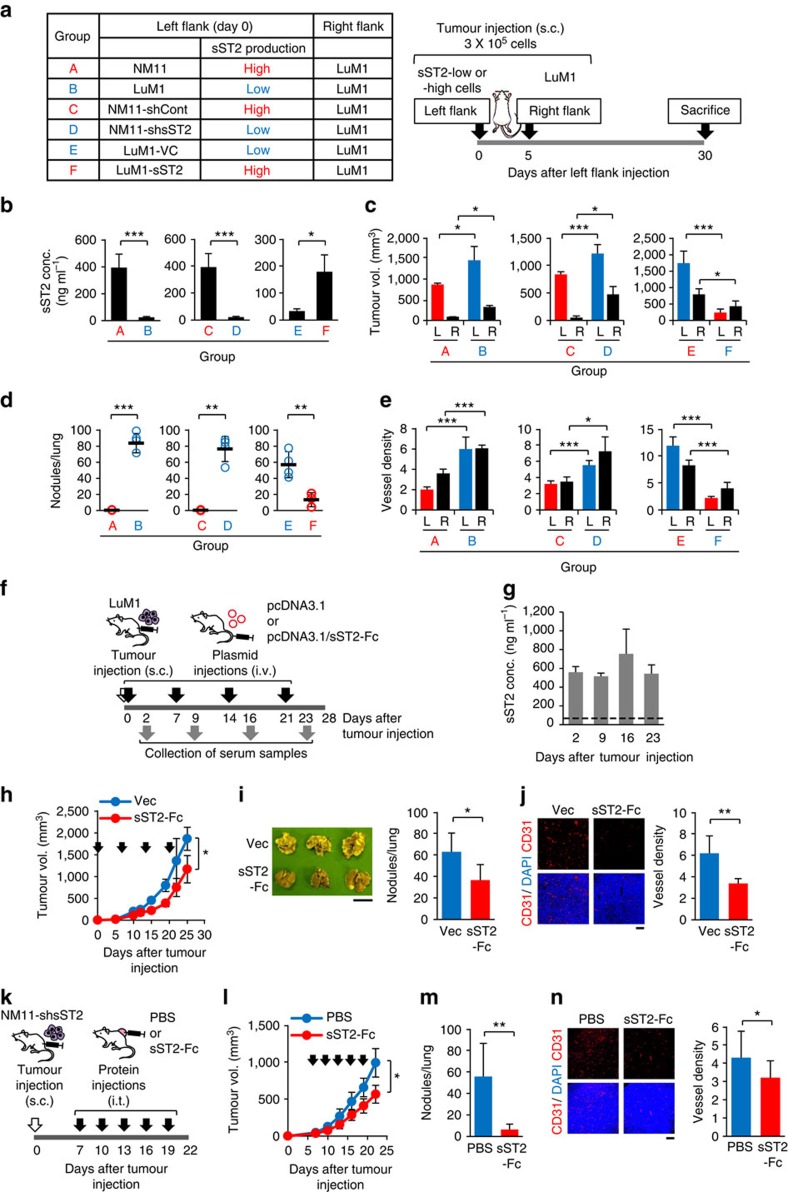
Circulating and recombinant sST2 suppresses the malignant growth of LuM1 tumours. (**a**) Injected CRC cell pairs (group A-F) and schematic procedure of the injection of tumour cells. BALB/c mice were subcutaneously inoculated with sST2 high-expressing cells (NM11 or LuM1-sST2) or low-expressing cells (LuM1 or NM11-shsST2) into the left flank (L) on Day 0, followed by the injection of LuM1 cells into the right flank (R) on Day 5. The mice (*n*=6 mice per group) were killed on Day 30, and the blood, tumours and lungs were collected. (**b**) sST2 serum levels. (**c**) Tumour volume. (**d**) Number of lung metastatic nodules. (**e**) Vessel density in the tumours examined by CD31 staining (*n*=15 fields). The data are shown as the mean±s.d. (**f**) Protocol used for the tumour inoculation and hydrodynamic injection of the sST2-Fc-expressing plasmid (*n*=6 mice per group). The mice were killed and the tumours and lungs were removed on Day 28. (**g**) Monitoring of sST2 serum concentrations by ELISA. Serum samples were collected at the time points indicated in (**f**). The dashed line indicates the mean value of sST2 serum concentration in non-tumour-bearing age-matched BALB/c mice (17.9 ng ml^−1^) (*n*=5). (**h**) Tumour growth. Arrows denote the plasmid injection time points. (**i**) Pulmonary metastasis. Scale bar: 1 cm. (**j**) CD31 staining of cryostat sections of tumours (left) and tumour vessel density (right) (*n*=15 fields). Scale bar: 100 μm. (**k**) Protocol used for intratumoural (i.t.) administration of the sST2-Fc protein (100 μg) into NM11-shsST2 tumours (*n*=7 mice per group). (**l**) Tumour growth. Arrows denote the protein injection time points. (**m**) Pulmonary metastasis. (**n**) Angiogenesis. CD31 staining of cryostat sections of tumours (left) and tumour vessel density (right) (*n*=39∼40 fields). Scale bar: 100 μm. The data are shown as the mean±s.d. *P<0.05, **P<0.01, ***P<0.001 by Student's *t*-test.

Notably, sST2 serum concentrations appeared to correlate with tumour size in mice bearing sST2 high-expressing tumours (NM11-shCont tumours and LuM1-sST2 tumours) and remained at basal levels in mice bearing sST2 low-expressing tumours (LuM1-VC tumours and NM11-shsST2 tumours) (Supplementary Fig. 3). We then investigated whether circulating sST2 influenced the growth, metastasis and angiogenesis of LuM1 tumours *in vivo* using two different methods. First, mice were subcutaneously inoculated with sST2 high- or low-expressing cells into the left flank on Day 0, followed by the injection of LuM1 cells into the right flank on Day 5. In addition to the quantification of sST2 in the serum, growth and angiogenesis were examined in LuM1 tumours and lung metastases on Day 30 (Fig. 4a). High sST2 serum levels were detected only when sST2 high-expressing tumours were present on the left flank (Fig. 4b). Interestingly, tumour growth, angiogenesis and metastasis were obviously reduced only when sST2 high-expressing tumours (NM11, NM11-shCont and LuM1-sST2) were present on the left flank (Fig. 4c–e, Supplementary Fig. 4), implying that sST2 released by sST2-high expressing tumours into the blood suppressed the malignant phenotypes of the distant LuM1 tumours.

To obtain more direct evidence, we next examined whether circulating recombinant sST2 had inhibitory effects on the tumour growth and metastasis of LuM1 cells. For this purpose, we constructed a plasmid expressing an sST2-Fc fusion protein and transfected it into HEK293T cells. The cells efficiently secreted the sST2-Fc fusion protein into the culture supernatant (Supplementary Fig. 5a,b). Then, we hydrodynamically injected the plasmid into the tail veins of LuM1 tumour-bearing mice weekly (Fig. 4f). sST2-Fc serum levels were dramatically increased after a single injection of the plasmid but returned to basal levels by Day 3 post-injection (Supplementary Fig. 5c). However, when the mice received weekly injections of the plasmid, a high level of sST2-Fc in the serum could be achieved after each injection (Fig. 4g). Remarkably, we observed a significant reduction of tumour growth, pulmonary metastasis and angiogenesis of LuM1 tumours (Fig. 4h–j). These data clearly indicate that circulating sST2-Fc can inhibit the malignant growth of CRC. Although sST2-Fc seems to be weaker in suppressing the malignant growth of CRC cells than tumour cell-derived native sST2, it may be due to its lower affinity to IL-33 or a shortage of local accumulation of the protein in the tumour tissues.

From a therapeutic point of view, we also tested the effect of an intratumourally administered sST2-Fc fusion protein on the malignant growth of CRC. To this end, we prepared bioactive glycosylated form of the sST2-Fc protein, which was secreted by EpiCHO-S cells transfected with the pcDNA3.1-sST2-Fc plasmid (Supplementary Fig. 6), and administered 100 μg of the protein intratumourally every 3 days starting from day 7 post-injection of the NM11-shsST2 cells (Fig. 4k). Again, tumour growth, metastasis and angiogenesis were significantly suppressed (Fig. 4l–n). These results indicate that extracellular administration of the sST2-Fc protein can reverse the malignant growth of cells expressing *sST2* shRNA.

### sST2 inhibits IL-33-induced angiogenesis

**Figure 5:**
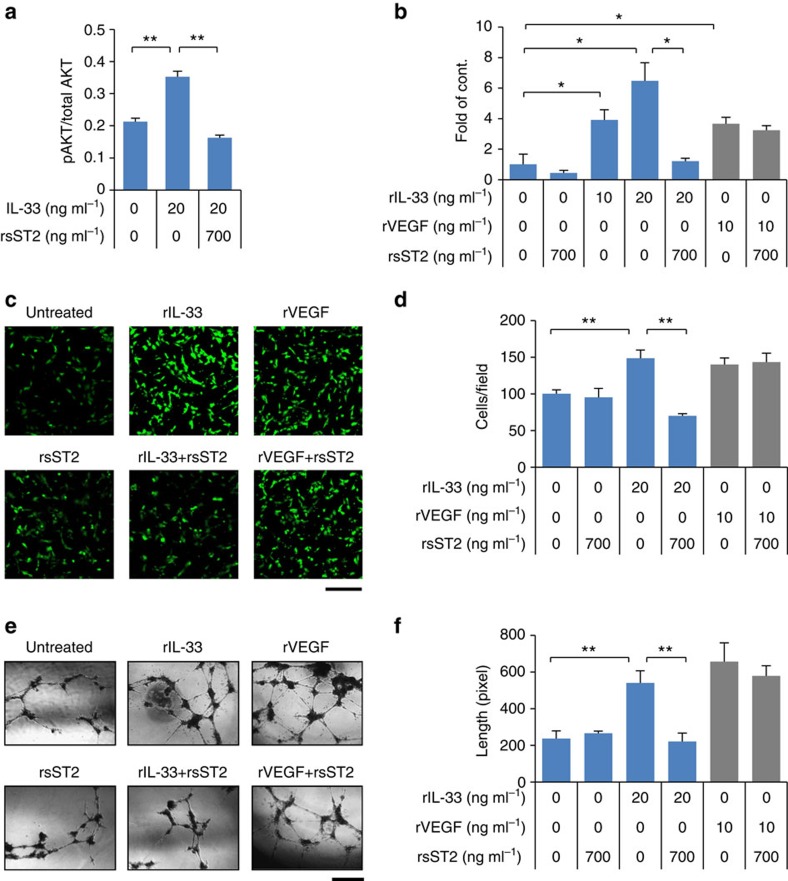
sST2 inhibits the IL-33-induced angiogenic response of HUVECs. (**a**) Akt activation. HUVECs were treated with rIL-33 (20 ng ml^−1^) in the presence or absence of rsST2 (700 ng ml^−1^). After 30 min of incubation, Akt phosphorylation levels were analysed and presented as the relative ratio of phospho-Akt to total Akt (*n*=3). (**b**) Cell proliferation. HUVECs were treated with or without rIL-33 (10 or 20 ng ml^−1^) or rVEGF (10 ng ml^−1^) in the presence or absence of rsST2 (700 ng ml^−1^) for 36 h. Cell proliferation was measured by BrdU incorporation (*n*=3). (**c**,**d**) Cell migration. HUVEC cell migration was assessed using FluoroBlok (8-μm pore size) (*n*=15 fields). The lower surface of the filter was coated with 10 μg of gelatin. M199 medium containing rIL-33 (20 ng ml^−1^) or rVEGF (10 ng ml^−1^) with or without rsST2 (700 ng ml^−1^) was placed in the lower wells. Migrated cells were stained with Calcein AM. Scale bar: 200 μm. (**e**,**f**) Tube formation. HUVECs were treated with or without rIL-33 (20 ng ml^−1^) or rVEGF (10 ng ml^−1^) in the presence or absence of rsST2 (700 ng ml^−1^) (*n*=9 fields). Scale bar: 50 μm. All experiments were performed in triplicate. The data are shown as the mean±s.d. *P<0.01, **P<0.001 by Student's *t*-test. Data are representative of three independent experiments.

As demonstrated above, sST2 suppressed tumour growth *in vivo* but had no effect on cellular proliferation *in vitro*, suggesting that sST2 might function by acting on tumour stromal cells. Based on the observations that IL-33 exhibited a pro-angiogenic effect in human umbilical vein endothelial cells (HUVECs) via Akt signalling^29^ and that sST2 expression in human and mouse CRC cells resulted in decreased tumour vessel density (Figs 1, 2, 3, 4), we investigated the involvement of sST2 in the inhibition of IL-33-mediated angiogenic responses of HUVECs. The expression of ST2L and IL-1RAP was confirmed in HUVECs at the mRNA and protein levels (Supplementary Fig. 7), which was consistent with a previous report^30^. Then, we treated HUVECs with rIL-33 alone or in combination with rsST2 and determined the ratio of phospho-Akt/total Akt. rIL-33 promoted Akt phosphorylation, and phosphorylation was decreased by co-treatment with rsST2 (Fig. 5a). Similarly, a thymidine incorporation assay (Fig. 5b), migration assay (Fig. 5c,d) and tube formation assay using a two-dimensional Matrigel (Fig. 5e,f) revealed the inhibitory effect of rsST2 on rIL-33-induced responses of HUVECs. Moreover, rsST2 inhibited the rIL-33-induced accumulation of haemoglobin in the plugs in an *in vivo* Matrigel plug assay (Supplementary Fig. 8a,b) and retarded the rIL-33-induced endothelial cell sprouting in an aortic ring assay (Supplementary Fig. 8c,d). Together, these data indicate that sST2 suppresses IL-33-induced angiogenic responses *in vitro* and *in vivo*. Notably, vascular endothelial growth factor (VEGF) production by tumour cells and its concentrations in tumour tissues were comparable irrespective of sST2 expression (Supplementary Fig. 9), and rsST2 treatment had no effect on VEGF-induced angiogenic responses (Fig. 5b–f).

Because the effect of sST2 on tumour angiogenesis was remarkable, we examined the role of VEGF in the current tumour model. For this, we administered neutralizing rat anti-mouse VEGF antibody (clone 2G11-2A05)^31^ or isotype-matched control IgG (100 μg/mouse) into mice bearing NM11-shsST2 and LuM1-VC tumours. The results showed that the anti-VEGF antibody caused a decrease in microvessel density (Supplementary Fig. 10a). Thus, both VEGF and IL-33 are substantially involved in and probably cooperate closely in inducing tumour angiogenesis in CRC. Perhaps surprisingly, neutralization of VEGF was not sufficient to repress tumour growth (Supplementary Fig. 10b). We do not know the exact reason for this, but staining of the tumour tissues for NG2, a pericyte marker, revealed that the antibody increased the pericyte coverage of CD31-positive tumour vessels (Supplementary Fig. 10c). This suggests the normalization of vessel functionality and may possibly explain the limited repression of tumour growth by anti-VEGF antibody despite the decrease in vessel density.

### sST2 inhibits Th1/Th2 response in tumours

**Figure 6:**
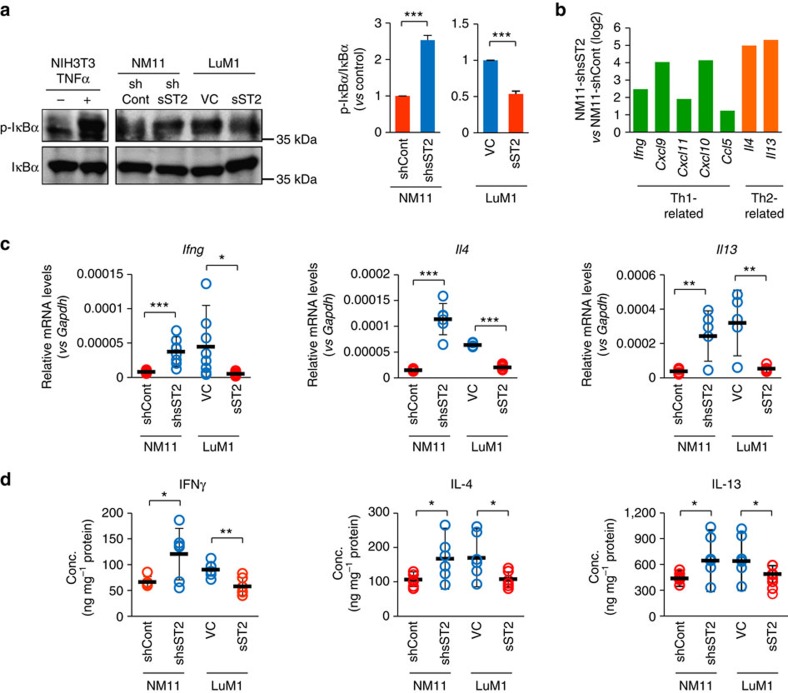
Tumour-derived sST2 suppresses the expression of Th1- and Th2-related genes in tumours. (**a**) IκBα phosphorylation in the tumours. The lysates of NM11-shCont, NM11-shsST2, LuM1-VC and LuM1-sST2 tumours were subjected to immunoblot analysis of p-IκBα and total IκBα (*n*=3). The lysates of NIH3T3 cells treated with TNF-α (10 ng ml^−1^) were used as a positive control. (**b**) Upregulation of Th1- and Th2-related genes in NM11-shsST2 tumours. Gene expression profiling of cytokines and chemokines between NM11-shsST2 and NM11-shCont tumours was performed using a PCR array. (**c**) qRT-PCR analysis of *Ifnγ*, *Il4* and *Il13* mRNA expression in the indicated tumours (*n*=7). (**d**) Concentrations of IFN-γ, IL-4 and IL-13 in the indicated tumours (*n*=7). The data are shown as the mean±s.d. *P<0.05, **P<0.01, ***P<0.001 by Student's *t*-test.

IL-33 enhances the production of both pro- and anti-inflammatory cytokines from ST2L-expressing inflammatory cells, including Th2 lymphocytes and mast cells, primarily via NF-κB signalling^32^,^33^. Therefore, IL-33 released from tumours may activate the NF-κB signalling pathways in tumour-infiltrating inflammatory cells, which in turn may be attenuated by sST2. Prior to testing this possibility, we examined whether IL-33 was present in tumour tissues. Indeed, abundant IL-33 proteins were detected in mouse CRC tumours compared with normal tissues (colon, small intestine, liver, spleen and lung) (Supplementary Fig. 11a) irrespective of sST2 expression levels in the tumour cells (Fig. 3a). Pro-IL-33 is actively secreted into the extracellular milieu as an ‘alarmin' by inflammatory cells, such as macrophages and mast cells, and by epithelial cells, myofibroblasts, fibroblasts, adipocytes, endothelial cells and smooth muscle cells. This pro-IL-33 is primarily localized in the nucleus and then processed into its mature form^32^,^33^,^34^. We could detect both pro-IL-33 and mature IL-33 in tumour lysates (Supplementary Fig. 11b). As expected, IκBα phosphorylation was higher in sST2 low-expressing tumours (LuM1-VC and NM11-shsST2 tumours) than in sST2 high-expressing tumours (LuM1-sST2 and NM11-shCont tumours) (Fig. 6a). These findings also imply that sST2 affects the expression of NF-κB-targeted cytokines and chemokines in tumours. Then, we compared the cytokine and chemokine gene expression profiles between the NM11-shsST2 and NM11-shCont tumours using a PCR Array platform. Among the 84 genes examined, 31 genes were upregulated and 13 genes were downregulated more than two-fold in the NM11-shsST2 tumours compared with the NM11-shCont tumours (Supplementary Fig. 12); some of these genes were direct targets of NF-κB (Supplementary Table 1). Notably, both of the representative Th2 cytokine genes *Il4* and *Il13* and the representative Th1 cytokine gene *Ifng* were significantly upregulated in NM11-shsST2 tumours (Fig. 6b). These changes were confirmed at the mRNA and protein levels by qRT-PCR and enzyme-linked immunosorbent assay (ELISA) (Fig. 6c,d). Interestingly, the expression of these cytokines was lower in LuM1-sST2 tumours than in VC-sST2 tumours (Fig. 6c,d), which represented an inverse association with the sST2 expression level. Thus, sST2 seemed to inhibit both Th1 and Th2 responses induced by IL-33 in the tumour microenvironment.

### sST2 inhibits macrophage infiltration and polarization

**Figure 7:**
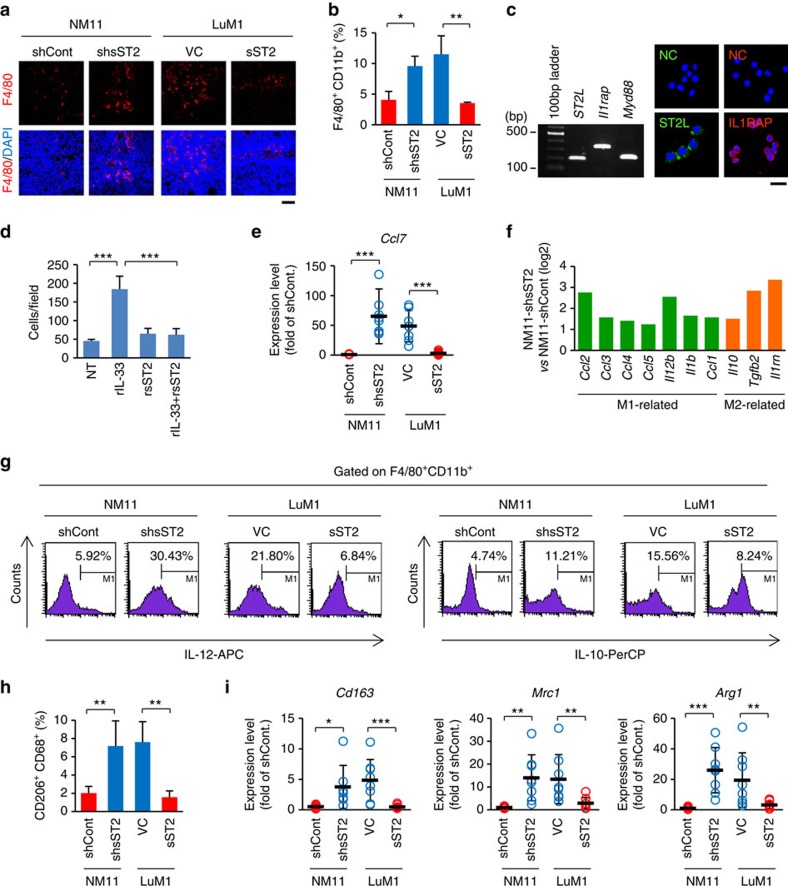
Tumour-derived sST2 inhibits macrophage infiltration and M2a polarization. (**a**) F4/80^+^ cells in the tumours. Cryostat sections of the indicated tumours were stained for F4/80. Scale bar: 50 μm. (**b**) The percentage of F4/80^+^CD11b^+^ cells in the tumours. F4/80^+^CD11b^+^ cells in the tumours were analysed by FACS (*n*=3). (**c**) ST2L, Il1rap and Myd88 expression in murine macrophage RAW264.7 cells. Left, RT-PCR analysis. Right, Immunofluorescent staining. NC, negative control (second antibody only). Scale bar: 20 μm. (**d**) Inhibition of RAW264.7 cell migration by rsST2 in response to rIL-33 (*n*=3). The cells were treated with rIL-33 (20 ng ml^−1^) in the presence or absence of rsST2 (700 ng ml^−1^). (**e**) qRT-PCR analysis of *Ccl7* expression in the indicated tumours (*n*=8). (**f**) Upregulation of M1- and M2-related genes in NM11-shsST2 tumours. Gene expression profiling of cytokines and chemokines between NM11-shsST2 and NM11-shCont tumours was performed using a PCR array. (**g**) FACS analysis of IL-12 and IL-10-expressing cells in the F4/80^+^CD11b^+^ population. The cells first gated on F4/80^+^CD11b^+^ were further analysed for IL-12 or IL-10. (**h**) FACS analysis of CD206^+^CD68^+^ cells in the indicated tumours (*n*=4). (**i**) qRT-PCR analysis of *Cd163*, *Mrc1* and *Arg1* mRNA in the indicated tumours (*n*=8). The data are shown as the mean±s.d. *P<0.05, **P<0.01, ***P<0.001 by Student's *t*-test.

Immunofluorescence staining showed that F4/80^+^ macrophages accumulated in sST2 low-expressing tumours (NM11-shsST2 and LuM1-VC) at higher levels compared to sST2 high-expressing tumours (NM11-shCont and LuM1-sST2) (Fig. 7a). Fluorescence-activated cell sorting (FACS) analysis also confirmed a larger number of F4/80^+^CD11b^+^ macrophages in the sST2 low-expressing tumours (Fig. 7b, Supplementary Fig. 13a). These data suggested that sST2 might play an inhibitory role in IL-33-induced macrophage infiltration of the tumour stroma. To address this possibility, we used murine macrophage RAW264.7 cells that express ST2L, IL1RAcP and MyD88 (Fig. 7c). Indeed, rIL-33 promoted the migration of RAW264.7 cells in a manner that was inhibited by rsST2 (Fig. 7d). Thus, it is likely that extracellular IL-33 in tumour tissues stimulates macrophage infiltration of the tumour stroma, and sST2 interferes with this process. In support of this result, expression of the *Ccl7* (Chemokine (C-C Motif) Ligand 7) gene, which encodes monocyte chemotactic protein 3 produced by macrophages that strongly attracts monocytes during inflammation and metastasis^35^,^36^, was significantly upregulated in NM11-shsST2 and LuM1-VC tumours compared with NM11-shCont and LuM1-sST2 tumours (Fig. 7e).

Naïve monocytes differentiate into M1 and M2 macrophages^35^. Generally, tumour-associated macrophages (TAMs) acquire an M2-polarized phenotype and promote angiogenesis and metastasis and suppress adaptive immunity through the expression of cytokines, chemokines, growth factors and matrix metalloproteases^37^,^38^. Our PCR array data showed that both M1-related genes (*Ccl2*, *Ccl3*, *Ccl4*, *Ccl5*, *Il12b*, *Il1b* and *Ccl1*) and M2-related genes (*Il10, Tgfb2* and *Il1rn*) were significantly upregulated in NM11-shsST2 tumours compared with NM11-shCont tumours (Fig. 7f). In accordance with this finding, FACS analysis showed that both M1 (IL-12^+^) and M2 (IL-10^+^) macrophages were increased in sST2 low-expressing tumours compared with sST2 high-expressing tumours (Fig. 7g). This result suggests that tumour-derived sST2 suppresses both M1 and M2 polarization, likely due to the reduction in Th1 and Th2 responses by sST2 described above.

M2 macrophages are further classified into at least four subsets: M2a, induced by IL-4 or IL-13; M2b, induced by immune complexes and agonists of toll-like receptors (TLRs) or IL-1 receptors; M2c, induced by IL-10, TGF-β and glucocorticoid hormones; and M2d, induced by IL-6 and adenosines^39^,^40^,^41^,^42^. Because production of IL-4 and IL-13 was markedly upregulated in sST2 low-expressing tumours (Fig. 6c,d), we assumed that the proportion of the M2a subset was increased in these tumours. As expected, FACS analysis showed an increase in CD206^+^CD68^+^ M2a phenotype cells in sST2 low-expressing tumours (Fig. 7h, Supplementary Fig. 13b). Consistent with this finding, expression levels of the M2a markers *Cd163*, *Mrc1*, and *Arg1* were high in sST2 low-expressing tumours (Fig. 7i). Together, these results suggest that sST2 reduces IL-33-induced macrophage infiltration of the tumour stroma and M2a polarization of infiltrated macrophages.

## Discussion

The present results show that mouse CRC tissues contain much higher levels of IL-33 than normal tissues, such as the colon. Full-length IL-33 secreted by inflammatory cells or released from damaged cells is biologically active but is processed by neutrophil elastase and cathepsin G to generate more active mature forms (two major products of approximately 21 and 18 kDa and one major product of ∼20 kDa, respectively)^43^. Because we detected only one major processed band (approximately 18 kDa) in mouse CRC tumours, full-length IL-33 may be processed by elastase, thereby making the tumour microenvironment more inflammatory and enhancing CRC growth and metastasis. Indeed, recent reports have shown increased expression of IL-33 in the cancerous epithelium compared with the adjacent normal epithelium, and a correlation has been reported between IL-33 expression and a poor prognosis in CRC patients^22^. A recent immunohistochemical study showed that the ST2 expression level in CRCs was associated with TNM stages but not with overall survival^24^. In contrast, our results showed that there was no correlation between the TNM stages and the ST2 IHC scores. Although the reason for this discrepancy is unclear, we speculate that this is attributed to limited sizes of tumour lesions on the tissue arrays and the difficulty of discrimination between ST2L and sST2. However, intriguingly, we observed an association between low ST2 IHC scores and greater microvessel density. Furthermore, we found that ST2, probably sST2, was expressed in cancerous cells in primary tissues but not in liver metastatic lesions from CRC patients, and the serum sST2 level was lower in CRC patients in more advanced CRC stages. Although the serum levels were lower in humans than in mice, we suppose that large amounts of sST2 exist in the tumour microenvironment in human CRC patients, judging from the intensity of IHC. Together, these results strongly indicate that IL-33/ST2L axis is involved in CRC progression.

Here, we focused on tumour-derived sST2. We assumed that its suppressive effect on CRC malignancy occurred through modification of the inflammatory tumour microenvironment. Our results clearly demonstrate that knockdown of sST2 in non-metastatic cells enhances tumour growth, metastasis and angiogenesis, whereas its overexpression in high-metastatic cells suppresses these phenotypes. Furthermore, we present evidence that circulating sST2 (including both tumour-derived sST2 and sST2-Fc) and intratumourally administered recombinant sST2-Fc protein suppress the growth and metastasis of CRC. However, we could not exclude the possibility of Fc-dependent cellular cytotoxicity of the Fc fusion proteins. Nonetheless, these results suggest that sST2 may function as an anti-cancer and anti-metastatic agent following systemic administration in CRC patients. Additional studies (especially in immunological aspects) are required to address this issue because an imbalance of sST2 and the IL-33/ST2L axis could trigger various inflammatory disorders.

Insights into the mechanisms by which sST2 suppress the malignant growth of CRC cells revealed that it inhibited the IL-33-induced angiogenic response of endothelial cells. Because angiogenesis was suppressed in sST2 high-expressing tumours, IL-33 might be an important proangiogenic factor in CRC. However, we could not rule out the possibility that sST2 inhibited tumour angiogenesis independent of IL-33 by suppressing the production of other proangiogenic factors by inflammatory cells and TAMs. In 4T1 breast cancer model mice, intraperitoneal administration of IL-33 promoted tumour angiogenesis through facilitating the intratumoural accumulation of immunosuppressive and innate lymphoid cells; this process was attenuated in ST2-deficient mice^21^. However, because CD45^+^CD3^+^ lymphoid cells were not significantly decreased in sST2-expressing tumours (Supplementary Fig. 13c), the involvement of innate lymphoid cells in angiogenesis could be excluded in our mouse model.

NF-κB may contribute to CRC progression by regulating the expression of diverse target genes involved in cell proliferation, angiogenesis, metastasis, and immune and inflammatory responses^44^. The IL-33/ST2L axis promotes the expression of Th2-associated cytokines in Th2 cells through NF-κB activation^30^ and is specifically suppressed by sST2^45^. From these reports, we reasoned that tumour-derived sST2 could modulate the inflammatory tumour microenvironment through interference with IL-33-induced NF-κB signalling. As expected, NF-κB activation (as assessed by measuring IκB-α phosphorylation and expression levels of the representative Th2 cytokines IL-4 and IL-13) was significantly downregulated in sST2-expressing tumours compared with sST2-deficient tumours. Interestingly, we also found that Th1-cytokines were downregulated. Although previous studies showed that sST2 suppressed IL-33-induced Th2 differentiation^46^ and Th1 to Th2 skewing^47^, a recent *in vitro* study demonstrated that IL-33, in cooperation with other pro-inflammatory cytokines, could amplify both Th1- and Th2-oriented immune responses through its interactions with basophils, Th2 cells, and iNKT and NK cells^48^. Therefore, IL-33 may promote both Th1 and Th2 responses in the complicated tumour microenvironment, wherein various pro-inflammatory cytokines are abundant. As such, tumour-derived sST2 may interfere with IL-33-induced Th1 and Th2 responses in our model, which in turn may contribute to the anti-growth and anti-metastatic effects of sST2 in immunocompetent mice.

TAMs are notorious for enhancing tumour malignancy by supporting tumour angiogenesis, tumour cell invasion, migration, and intravasation, and the suppression of anti-tumour immune responses^35^,^36^,^37^,^38^,^39^. TAM accumulation is associated with a poor prognosis in a variety of human cancers, including CRC^49^. Interestingly, we found that TAMs were small in number in sST2 high-expressing tumours, suggesting that sST2 inhibited the IL-33-induced recruitment of monocytes or the attraction of marginal macrophages into tumour tissues. In support of this finding, we found that IL-33 enhanced the cell motility of RAW264.7 cells in a manner that was inhibited by sST2. IL-33 has been proposed to promote M2 but not M1 polarization through promoting Th2 responses^36^,^37^,^38^. However, a recent study showed that IL-33 primarily induced the production of M1 chemokines in bone marrow-derived naïve macrophages and promoted or amplified the expression of M2 chemokines and M2 markers in M1- and/or M2-polarized macrophages^8^. Consistent with this report, our PCR array analysis revealed a significant upregulation of both M1-related genes (*Ccl2*, *Ccl3*, *Ccl4*, *Ccl5*, *Il12b*, *Il1b* and *Ccl1*) and M2-related genes (*Il10, Tgfb2* and *Il1rn*) in sST2 low-expressing tumours. This finding suggests that tumour-derived sST2 attenuates IL-33-induced macrophage polarization in both directions. Indeed, our FACS analysis showed that both the IL-12^+^ M1 and IL-10^+^ M2 populations were increased in sST2 low-expressing tumours. Nevertheless, the expression of M2a markers (that is, CD163 (*Cd163*), mannose receptor complex-1 (*Mrc1*) and arginase-1 (*Arg1*) was significantly upregulated in sST2 low-expressing tumours. Taken together, these results suggest that sST2 may inhibit IL-33-induced monocyte infiltration and M2a polarization of the infiltrated cells, thereby reducing the stimulatory effects of TAMs on tumour angiogenesis, invasion and metastasis.

In conclusion, we demonstrate herein that sST2 can suppress tumour malignant growth locally and systemically by inhibiting IL-33-induced tumour angiogenesis and by modifying the tumour microenvironment. This study highlights the possibility of using recombinant sST2 as an anti-tumourigenic and anti-metastatic agent for CRC. Further studies are warranted to explore the present findings in greater detail.

## Methods

### Reagents

Human recombinant IL-33 (rIL-33) and the human recombinant ST2/IL-1 R4 Fc Chimera Protein (rsST2) were purchased from R&D Systems, Inc. (Minneapolis, MN, USA). Human recombinant VEGF (rVEGF) was provided by MBL (Nagoya, Japan).

### Cells and cell culture

Human SW480 and SW620 colon adenocarcinoma cells derived from primary and secondary tumours resected from a single patient, respectively, were obtained from ATCC (Manassas, VA, USA). Low-metastatic NM11 and high-metastatic LuM1 cells were established from the murine colon adenocarcinoma Colon 26 cell line^50^ and supplied by Dr S. Shimizu, Aichi Cancer Center, Nagoya, Japan These cells were cultured in Dulbecco's modified Eagle's medium containing 10% fetal bovine serum (FBS). HUVECs were purchased from Lonza Walkersville, Inc. (Walkersville, MD, USA) and grown in endothelial basal medium (EBM) supplemented with EBM SingleQuots (Lonza), FBS, hEGF, hydrocortisone, GA-1000 and BBE. The murine macrophage cell line RAW264.7 was kindly supplied by Dr M. Nakamura, Collaboration Center, Shimane University, Izumo, Japan. These cells were maintained at 37 °C under 5% CO_2_. ExpiCHO-S cells were purchased from Gibco, Thermo Fisher Scientific Inc. (Waltham, MA, USA) and cultured in ExpiCHO expression medium at 37 °C under 8% CO_2_ with shaking in an Erlenmeyer flask. All the cell lines were not authenticated after purchase or transferred from other laboratories (propagated and stored in liquid N_2_ after receipt). All cell lines were free of mycoplasma contamination as assessed by e-Myco Mycoplasma PCR Detection Kit (Cosmo Bio Co Ltd., Tokyo, Japan).

### Immunohistochemistry

Archivally matched, paraffin-embedded primary and liver metastasis tissue samples from patients with CRC were cut into 4-μm sections. The use of the pathologic samples was approved and reviewed by the Ethics Committee of Shimane University Hospital (Approval no. 1348). Colon Adenocarcinoma Tissue Arrays were purchased from US Biomax, Inc., Rockville, MD, USA (35 women, mean age 58, range 24–78 and 35 men, mean age 57, range 27–76; 11 stage I, 36 stage II, 19 stage III and 4 stage IV). After antigen retrieval with proteinase K, the tissues were immunostained with a polyclonal anti-IL1RL1 antibody (Atlas Antibodies, HPA007406, 1:500) that reacts with both ST2L and sST2 or a monoclonal anti-human CD31 antibody (DAKO, IS610, 1:100) using an automatic immunostaining system (BenchMark XT, Ventana Medical Systems, Inc., Tucson, AZ, USA). To semi-quantitatively represent the ST2 immunostaining results, evaluable sections were classified into four IHC scores according to the percentage of ST2-positive cells within the total cancerous cells: IHC score 0, 0% positive; IHC score 1, 1–10% positive; IHC score 2, 11–50% positive; IHC score 3, >50% positive. The number of CD31-positive vessels was counted under a microscope.

For determination of vessel density in mouse tumours, tumours were surgically removed and immediately embedded and frozen in OCT compound. Cryostat sections (8-μm thick) were fixed in 4% paraformaldehyde for 10 min, blocked with 1% bovine serum albumin (BSA) in Dulbecco's phosphate-buffered saline (DPBS) and then incubated with rat anti-mouse monoclonal CD31 antibody (BD Biosciences, 550274, 1:100). After extensive washing with DPBS, sections were incubated with Alexa Fluor 594-conjugated goat anti-rat IgG (Invitrogen, Themo Fisher Scientific, A-11007, 1:300) for 1 h. Sections were counterstained with 4,6-diamidino-2-phenylindole and observed under a confocal laser scanning microscope (× 200 magnification) (Fluoview, Olympus). Pixel values of the CD31-positive areas were calculated for each image to determine the tumour vessel density using ImageJ software (National Institutes of Health). For this, background staining was excluded from images prior to isolating CD31-positive pixels of interest. Isolated positive pixels were then quantified. Positive pixel density was determined for 5–30 representative images per tumour. The primary antibodies used for IHC are shown in Supplementary Table 2.

### Immunocytochemistry

The cells were fixed with 4% formaldehyde and 5% sucrose in DPBS for 20 min, permeabilized with 0.5% Triton X-100 in DPBS for 5 min, and then blocked with 3% BSA/0.1% glycine in DPBS for 1 h. The cells were immunostained with primary antibodies for 1 h, washed with DPBS, and then incubated with secondary antibodies for 1 h^51^. The primary antibodies are shown in Supplementary Table 2. Alexa Fluor 488- and Alexa Fluor 594-conjugated species-specific antibodies (Invitrogen, 1:300) were used as secondary antibodies. The nuclei were counterstained with 4,6-diamidino-2-phenylindole (1 μg ml^−1^). Then, the slides were mounted with the Vectashield mounting medium (Vector Laboratories, Burlingame, CA, USA) and observed under a confocal laser scanning microscope (Fluoview, Olympus, Tokyo, Japan).

### sST2 level in the sera

The study was conducted according to the protocol for the collection of sera and was approved by the National Cancer Centre Hospital Ethics Committee (Approval no. 2010-096) in accordance with the Helsinki declaration. Patients with CRC (57 women, mean age 66.0, range 28–84 and 56 men, mean age 60.5, range 22–80) who had been diagnosed using TNM staging and underwent surgery from 2007 to 2013 were recruited in this study. The patients had given consent for their serum samples to be used in the future for the purpose of evaluation of diagnostic tests and new potential biomarkers. Serum sST2 levels were measured with a Human sST2 (soluble ST2) ELISA Kit (MBL).

### ST2 knockdown by shRNAs

The lentiviral transduction particles for sST2 knockdown were produced by transient co-transfection of HEK293T cells with the shRNA-encoding transfer vector, MISSION human *ST2* shRNA (TRCN0000058517) and mouse *ST2* shRNA (TRCN0000039054) in the pLKO.1-puro plasmid (Sigma-Aldrich Japan, Tokyo, Japan), and the compatible packaging plasmids (MISSION Lentiviral Packaging Mix). After 48 h incubation, the lentiviral particles were collected and stored at −80 °C until use. As controls, pLKO.1-puro control transduction particles (SHC001v) were used. SW480 cells or NM11 cells were transduced with the lentivirus stocks in the presence of 8 μg ml^−1^ polybrene overnight and then selected with 5 μg ml^−1^ puromycin for at least 2 weeks to establish control cells (shCont cells) or cells displaying stable sST2 downregulation (shsST2 cells).

### Construction of sST2 expression plasmids

Mouse *sST2* cDNA was amplified by PCR with 5′ and 3′ primers carrying appropriate restriction sites at the respective 5′ end using the total RNA isolated from the NM11 cells as a template. After electrophoresis on a 1% agarose gel, the PCR products were extracted from the gel using the QIAquick Gel Extraction Kit (Qiagen, Hilden, Germany), cloned into pGEM-TEasy (Promega, Fitchburg, WI, USA), digested with restriction enzymes and subcloned into pcDNA3.1 (Invitrogen), resulting in pcDNA3.1-sST2.

### RNA preparation and RT-PCR

Total RNA was extracted from cells and tumour tissues using TRI reagent (Sigma-Aldrich), and 1 μg of total RNA was reverse-transcribed with oligo (dT) primers and the M-MLV reverse transcriptase (Invitrogen). PCR was performed with the synthesized cDNA in a final volume of 25 μl containing 0.5 μM of each primer, 0.2 μM of the dNTP mixture, 2.5 mM MgCl_2_, 50 mM KCl, 10 mM Tris-HCl (pH 8.3) and 0.5 units of *Go*Taq (Promega). The PCR amplification was performed by incubating samples at 95 °C for 10 min, followed by the indicated cycles (94 °C for 1 min, 60 °C for 1 min and 72 °C for 1 min) and a final extension for 10 min at 72 °C. The amplified products were electrophoresed on a 1% agarose gel. The bands were stained with ethidium bromide and visualized under UV light. The PCR primer sequences are summarized in Supplementary Table 3.

### Quantitative RT-PCR (qRT-PCR)

Total RNA isolation and reverse transcription were performed as described above. Quantitative real-time PCR was done using THUNDERBIRD SYBR qPCR Mix (TOYOBO, Osaka, Japan) in a total volume of 20 μl on a Thermal Cycler Dice Real Time System TP860 (TaKaRa, Shiga, Japan): 95 °C for 1 min and 40 cycles of denaturation (95 °C for 15 s) and extension (60 °C for 1 min). Experiments were performed in triplicates. Following amplification, dissociation curve analyses were performed to confirm the amplicon specificity for each PCR run. The relative level of gene expression in human and mouse cells was normalized against human *GAPDH* or mouse *Gapdh*, respectively. Quantification was performed using the 2^−ΔΔCT^ method. The specific primer sets are shown in Supplementary Table 3.

### Real-time *Alu* PCR analysis

Human cells within the lungs of nude mice were detected by real-time *Alu* PCR essentially as previously described^52^. Whole lungs were harvested and genomic DNA was isolated using the DNeasy Tissue kit (Qiagen). Human tumour cells in the lungs were quantified using PCR-based detection of human *Alu* sequences and mouse genomic *Gapdh* sequences as a control. The primers used are summarized in Supplementary Table 3. Quantitative PCR was performed on 5 μg of genomic DNA as a template using the THUNDERBIRD SYBR qPCR Mix (TOYOBO) and 0.3 μM of the primers in a volume of 20 μl under the following conditions: 95 °C for 2 min and 30 cycles of 95 °C for 30 s, 65 °C for 20 s, and 72 °C for 20 s. Quantification of human DNA in mouse lung tissue was based on a standard curve constructed using serial dilutions (1:10) of human DNA isolated from SW620 cells and mouse DNA isolated from nude mouse lung tissue. For this, the cycle at which the *Alu* sequences became detectable (Ct*Alu*) was determined for each dilution series after PCR amplification, and then the relative level of *Alu* sequences normalized against *Gapdh* (ΔCt=(Ct*Alu*–Ct*Gapdh*)) was plotted against the relative amount of human DNA in the mouse DNA (%). The specific amplification of the *Alu* sequences was confirmed by the lack of amplification from 100% mouse DNA. Under these conditions, the detection limit of human DNA in mouse DNA was 0.001%.

### Tumour transplantation

All animal experiments were performed in compliance with the institutional guidelines for the care and use of animals in research. The protocol was approved by the IZUMO Campus Animal Care and Use Committee of Shimane University (Approval no: IZ26-7 and IZ27-37). SW480 and SW620 cells (1 × 10^6^ cells/mouse) were subcutaneously implanted with 50% Matrigel into 6-week-old female BALB/c nude mice (Japan SLC, Shizuoka, Japan) (*n*=7 mice per group). NM11 and LuM1 cells (3 × 10^5^ cells) were subcutaneously implanted into 6-week-old female BALB/c mice (CLEA Japan, Tokyo, Japan) (*n*=7 mice per group). For intratumoural administration of the sST2-Fc fusion protein, NM11-shsST2 cells (3 × 10^5^ cells) were subcutaneously injected into 6-week-old female BALB/c mice (*n*=14). One week after the injection, they were randomly (simple randomization) and blindly grouped into the control (*n*=7 mice) and the sST2-Fc groups (*n*=7 mice). Tumour volumes were evaluated by measuring two perpendicular diameters with calipers. The tumour volume (V) was calculated using the following equation: V=(*a*^2^ × *b*)/2, where *a* is the small diameter, and *b* is the large diameter. Attention was paid to tumour burden not to exceed 20% of the animal's body weight at the experimental endpoint of each metastasis assay (that is, 4,760 mm^3^ in a 25 g mouse, calculated from weight and density 1.05 g ml^−1^) that was allowed by our internal review board. For the orthotopic implantation model, the caecum of anesthetized mice with medetomidine (0.3 mg kg^−1^)/midazolam (4.0 mg kg^−1^)/butorphanol (5.0 mg kg^−1^) was exteriorized through a laparotomy, and cells (1.5 × 10^5^ cells in 50% Matrigel) were injected into the caecal wall between the mucosa and the muscularis externa layers using a 30-gauge needle attached to an insulin syringe (*n*=11, 8, 9 and 8 mice for NM11-shCont, NM11-shsST2, LuM1-VC and LuM1-sST2 cells, respectively). At the end of the experiments, the caecum and the liver were removed and examined macroscopically and histologically after haematoxylin-eosin staining. Mice were checked for their health once a day after tumour cell injection. All efforts including subcutaneous administration of analgesics were made to minimize pain and distress. Mice were euthanized by CO_2_ inhalation at the end of a study.

### Construction of sST2-Fc expression vector

The plasmid expressing the sST2-Fc fusion protein was constructed essentially according to the method described previously^53^. Briefly, cDNA fragment encoding IgG1 Fc was prepared by RT-PCR using total RNA isolated from human lymphocytes and the forward primer 5′-TCGGATCCATGGACAAAACTCACACATG-3′ and the reverse primer 5′-GCTCTAGAGCACTCATTTACCCGGGGACAG-3′. The resulting PCR product was subcloned into pGEM-T Easy vector and the insert was cut out with BamH1 and XbaI, gel-purified, and then inserted into the BamHI/XbaI cut pcDNA3.1 (Invitrogen) to make a plasmid pcDNA3.1-Fc. Next, *sST2* cDNA was amplified using total RNA isolated from NM11 cells and the forward primer 5′-ACAAGCTTGGCCGCCACCATGATTGACAGACAGAG-3′ and the reverse primer 5′-ACGGATCCGAGCAATGTGTGAGGGACACTCCT-3′. The amplified *sST2* cDNA was subcloned into pGEM-T Easy vector and the insert was cut out with HindIII and BamH1, gel-purified, and then inserted into the HindIII/BamHI cut pcDNA3.1-Fc to make a plasmid pcDNA3.1-sST2-Fc.

### Hydrodynamic tail vein injection

Hydrodynamic tail vein injections of the plasmid into 7-week-old female BALB/c mice were performed according to a previously described method^54^. Ten μg of the sST2-Fc expression plasmid (pcDNA3.1-sST2-Fc) and the empty plasmid were injected as 8% of the body weight volume (ml g^−1^) over 5–7 s.

### ELISA

For serum preparation, blood was allowed to clot and then centrifuged for 10 min at 1,000*g*. Serum was collected and immediately stored at −80 °C prior to use. For the preparation of conditioned media, the cells were seeded at a concentration of 1 × 10^6^ cells per well in a 24-well plate (BD Falcon, Franklin Lakes, NJ, USA) in 0.5 ml of Dulbecco's modified Eagle's medium containing 1% FBS. Conditioned media were harvested after 24 h of cultivation. For the preparation of tissue lysates, tissues were cut into pieces using a razor blade and were then placed in ice-cold DPBS containing cOmplete Protease Inhibitor Cocktail (Roche) and homogenized for 3 min at 30 Hz using a TissueLyser (QIAGEN). Supernatants were collected after centrifugation at 15,000*g* for 20 min at 4 °C. Human and mouse sST2 concentrations were measured with the Human sST2 (soluble ST2) ELISA Kit (MBL) and the Mouse ST2/IL-1 R4 Quantikine ELISA Kit (R&D Systems), respectively, according to the manufacturer's instructions. Mouse VEGF concentrations were measured with the Mouse VEGF Quantikine ELISA Kit (MBL). The concentrations of interferon-γ (IFN-γ), IL-4, IL-13 and IL-33 were measured with Ready-Set-Go ELISA kits (e-Bioscience, San Diego, CA, USA).

### Western blotting

Tumour tissues were cut into pieces using a razor blade. Necrotic tissue was removed and extracted with RIPA buffer (50 mM Tris-HCl, pH 7.4, 150 mM NaCl, 1% NP-40, 0.5% deoxycholate, 0.1% sodium dodecyl sulfate and 2 mM EDTA) containing the cOmplete Protease Inhibitor Cocktail and PhosSTOP Phosphatase Inhibitor Cocktail (Roche Applied Science, Penzberg, Upper Bavaria, Germany) on ice for 30 min. The lysates were centrifuged at 15,000*g* for 10 min at 4 °C, and the supernatants were used to estimate the amount of protein with the Bradford colorimetric assay using BSA as the standard. SDS-polyacrylamide gel electrophoresis and immunoblot analyses were performed according to our previously published standard method^51^. The primary antibodies used for immunoblot analysis are shown in Supplementary Table 2. The signals were visualized using ECL plus (GE Healthcare, Little Chalfont, UK). The membranes were scanned with an LAS4000 Luminoimaging Analyzer (GE Healthcare). Uncropped images were presented in Supplementary Fig. 14.

### Matrigel invasion assay

An invasion assay was performed using 8-μm pore size FluoroBlok Transwell chambers (BD Biosciences, Bedford, MA, USA) as previously described^51^, except that 5 ng ml^−1^ TGF-β was added to the lower chamber. The filters were coated with 30 μg of Matrigel (BD Biosciences). Cell invasion was evaluated by counting cells that migrated to the lower side of the filter under a confocal microscope (× 200) after staining with 1 μM Calcein AM (Molecular Probes, Thermo Fisher Scientific).

### Endothelial cell proliferation assay

HUVEC proliferation was evaluated by 5-bromo-2′-deoxyuridine (BrdU) incorporation using an ELISA kit (Cell Proliferation ELISA, BrdU, Roche Diagnostics, Basel, Switzerland) according to the manufacturer's instructions. HUVECs (1 × 10^4^) were seeded into a 96-well plate in M199 medium containing 1% FBS for serum starvation. After 24 h, the HUVECs were treated with rIL-33 (20 ng ml^−1^) alone or in combination with rsST2 for 36 h and then labelled with BrdU for 2 h. The cells were fixed and incubated with a peroxidase-conjugated anti-BrdU antibody. Then, the peroxidase substrate 3,3′,5,5′-tetramethylbenzidine was added, and BrdU incorporation was quantified by measuring the light emission of the samples using a luminometer.

### Endothelial cell migration assay

IL-33-induced motility of HUVECs was assessed using FluoroBlok (BD Biosciences) (8-μm pore size). Briefly, the lower surface of the filter was coated with 10 μg of gelatin. M199 medium containing 1% BSA and rIL-33 was placed in the lower wells. HUVECs were suspended at a final concentration of 10^6^ cells per ml in M199 medium containing 1% FBS. One hundred microlitres of the cell suspension was loaded into each of the upper wells, and the chamber was incubated at 37 °C for 4 h. Cell migration was evaluated by counting cells that migrated to the lower side of the filter under a confocal microscope (× 200) after staining with 1 μM Calcein AM.

### Tube formation assay

The wells of a 48-well plate were coated with 0.2 ml of growth factor-reduced Matrigel (BD Biosciences) by incubating for 30 min at 37 °C. HUVECs precultured in M199 medium containing 1% FBS for 6 h were harvested, resuspended in M199 medium and seeded onto the Matrigel layer at a cell density of 1 × 10^5^ cells/well. A designated concentration of rIL-33 and/or rsST2 was added to the medium. After 16 h of culture, tube formation was quantified by measuring the length of the capillary-like network on the photographs (× 40) using ImageJ software (National Institutes of Health).

### *In vivo* matrigel plug assay

Matrigel plug assay was performed according to the previously published method^55^. Briefly, 6-week-old BALB/c mice were injected subcutaneously with 0.5 ml of Matrigel containing the indicated amount of rIL-33 with or without rsST2 and 10 units of heparin. After 6 days, the skin of the mouse was pulled back to expose the Matrigel plug, which remained intact. The plugs were homogenized in DPBS, and total haemoglobin concentrations were measured by the Drabkin method using Hemokit-N (Nihon Shoji, Osaka, Japan) to quantify blood vessel formation. The haemoglobin concentration was calculated by comparison to a known amount of haemoglobin assayed in parallel.

### Aortic ring assay

The aortic ring assay was performed essentially as described^56^. Briefly, thoracic aortas were excised from 7-week-old male C57BL/6 mice (CLEA Japan) and sectioned into several pieces (aortic rings). Plates (48-well) were coated with 120 μl Matrigel. After gelling, the rings were placed in the wells. rIL-33 protein with or without rsST2-Fc was added to the wells in a final volume of 200 μl EBM. As a control, aortic rings in EBM alone were assayed. The plates were incubated at 37 °C, and the medium was changed every 2 days for 2 weeks. The angiogenic sprouting from the aortic rings (*n*=4) was photographed, and the sprouting was quantified by counting the number of vascular sprouts that directly originated from the aorta^29^.

### FACS analysis

To prepare single-cell tumour suspensions, tumours were minced on ice and digested in RPMI 1640 medium containing 1.33 mg ml^−1^ collagenase (Roche Diagnostics GmbH, Penzberg, Germany) and 100 U ml^−1^ DNase (Sigma-Aldrich) at 37 °C for 60 min. The digested tumour tissue was filtered through a 70-μm cell strainer (BD Biosciences). Single-cell suspensions were incubated with 10 μg ml^−1^ of anti-CD16/CD32 antibodies (BioLegend, San Diego, CA, USA) at 4 °C for 30 min for blocking. For intracellular staining, cells were incubated with antibodies against surface markers and then fixed and permeabilized with IntraPrep (BECKMAN COULTER, Brea, CA, USA) at room temperature in the dark for 20 min according to the manufacturer's instruction. Subsequently, cells were stained with the appropriate antibodies for 30 min at 4 °C in the dark and washed three times. Approximately 2 × 10^5^ cells were collected for each sample and analysed with a FACSCalibur flow cytometer (BD Biosciences). Populations of tumour macrophages (CD11b^+^F4/80^+^), M1 macrophages (IL-12^+^F4/80^+^CD11b^+^), M2 macrophages (IL-10^+^F4/80^+^CD11b^+^) and M2a-like macrophages (CD206^+^CD68^+^) were quantified using CellQuest Software (BD Biosciences). All antibodies used in the FACS analysis are summarized in Supplementary Table 2. Fluorescent-conjugated isotype control antibodies were used at a matching concentration to detect nonspecific binding to cells due to the antibody class.

### Preparation of sST2-Fc protein

ExpiCHO-S cells were transfected with the sST2-Fc expression plasmid (pcDNA3.1-sST2-Fc) using ExpiFectamine according to the manufacturer's protocol. After adding ExpiFectamine CHO Enhancer and ExpiCHO Feed, the cells were incubated at 32 °C in 5% CO_2_ for 12 days with shaking. The sST2-Fc fusion protein released into the medium was isolated using an nProtein A Sepharose 4 Fast Flow column (GE Healthcare Life Sciences, Buckinghamshire, UK) and dialysed against PBS. The purity of the sST2-Fc fusion protein was evaluated by SDS-PAGE followed by Coomassie brilliant blue staining and Western blotting. Glycosylation of the fusion protein was confirmed after treatment with glycopeptidase F (Takara Bio, Shiga, Japan). The biological activity of the protein was evaluated by the suppression of IkBα phosphorylation induced by rIL-33 (100 ng ml^−1^) in Lewis lung carcinoma P29 cells expressing functional ST2L^26^,^57^. This preparation was used for *in vivo* experiments.

### Statistical analysis

All data are presented as the mean±s.d. Statistical significance between data sets was tested by two-tailed Student's *t*-test with unpaired analysis. *P*<0.05 was considered significant. Sample sizes were chosen for each experiment based on pilot experiment examinations and sufficient statistic powers. All sample sizes were appropriate for assumption of normal distribution and variance was similar between compared groups.

### Data availability

The authors declare that the data supporting the findings of this study are available within the article and its Supplementary information files or from the corresponding author on reasonable request.

## Additional information

**How to cite this article:** Akimoto, M. *et al*. Soluble IL-33 receptor sST2 inhibits colorectal cancer malignant growth by modifying the tumour microenvironment. *Nat. Commun.* 7:13589 doi: 10.1038/ncomms13589 (2016).

**Publisher's note**: Springer Nature remains neutral with regard to jurisdictional claims in published maps and institutional affiliations.

## Supplementary Material

Supplementary Information Supplementary figures 1-14, Supplementary tables 1-3
