# Supplementary material for: Soluble IL-33 receptor sST2 inhibits colorectal cancer malignant growth by modifying the tumour microenvironment
Source: Nat Commun. 2016 Nov 24;7:13589. doi: 10.1038/ncomms13589 (PMC5123057; doi:10.1038/ncomms13589)
Supplement: Supplementary Information — Supplementary figures 1-14, Supplementary tables 1-3 [file ncomms13589-s1.pdf]

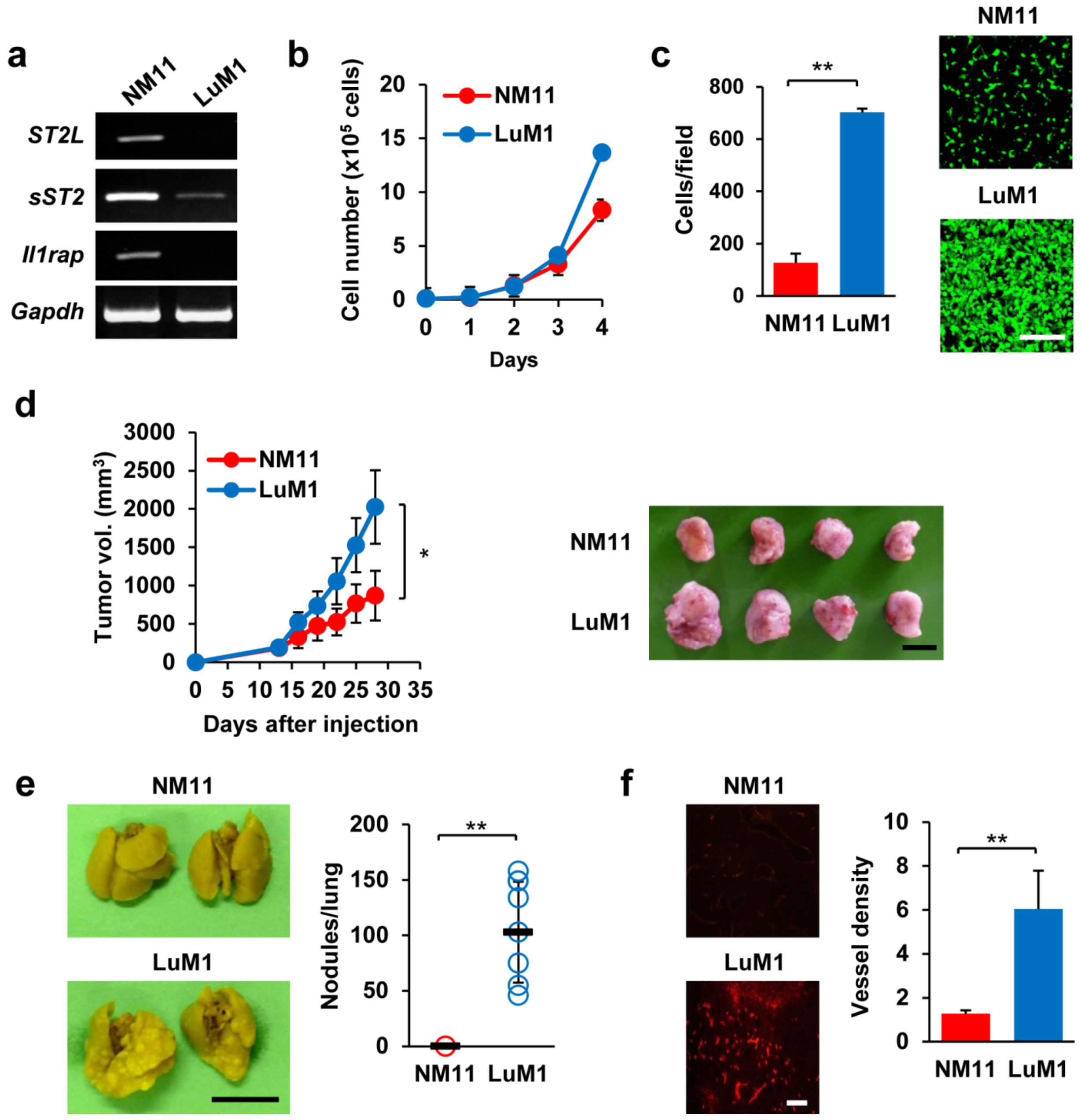

Supplementary Figure 1. sST2 expression is inversely correlated with the malignant growth of mouse CRC cells. (**a**) RT-PCR analysis of IL-33-related mRNA expression in NM11 and LuM1 cells. (**b**) Cell growth of NM11 and LuM1 cells *in vitro* (n=3). (**c**) Matrigel invasive ability of NM11 and LuM1 cells. Bar: 200 μm. (**d**) Growth of NM11 and LuM1 tumours in BALB/c mice (n=7 mice per group). Bar: 1 cm. (**e**) Spontaneous lung metastatic ability of NM11 and LuM1 cells (n=7 mice per group). Bar: 1 cm. (**f**) Vessel density in NM11 and LuM1 tumours. CD31 staining (left) and vessel density (right) (n=15 fields). Bar: 200 μm. The data are shown as the mean ± s.d.. *P<0.01, **P<0.001 by Student's t-test.

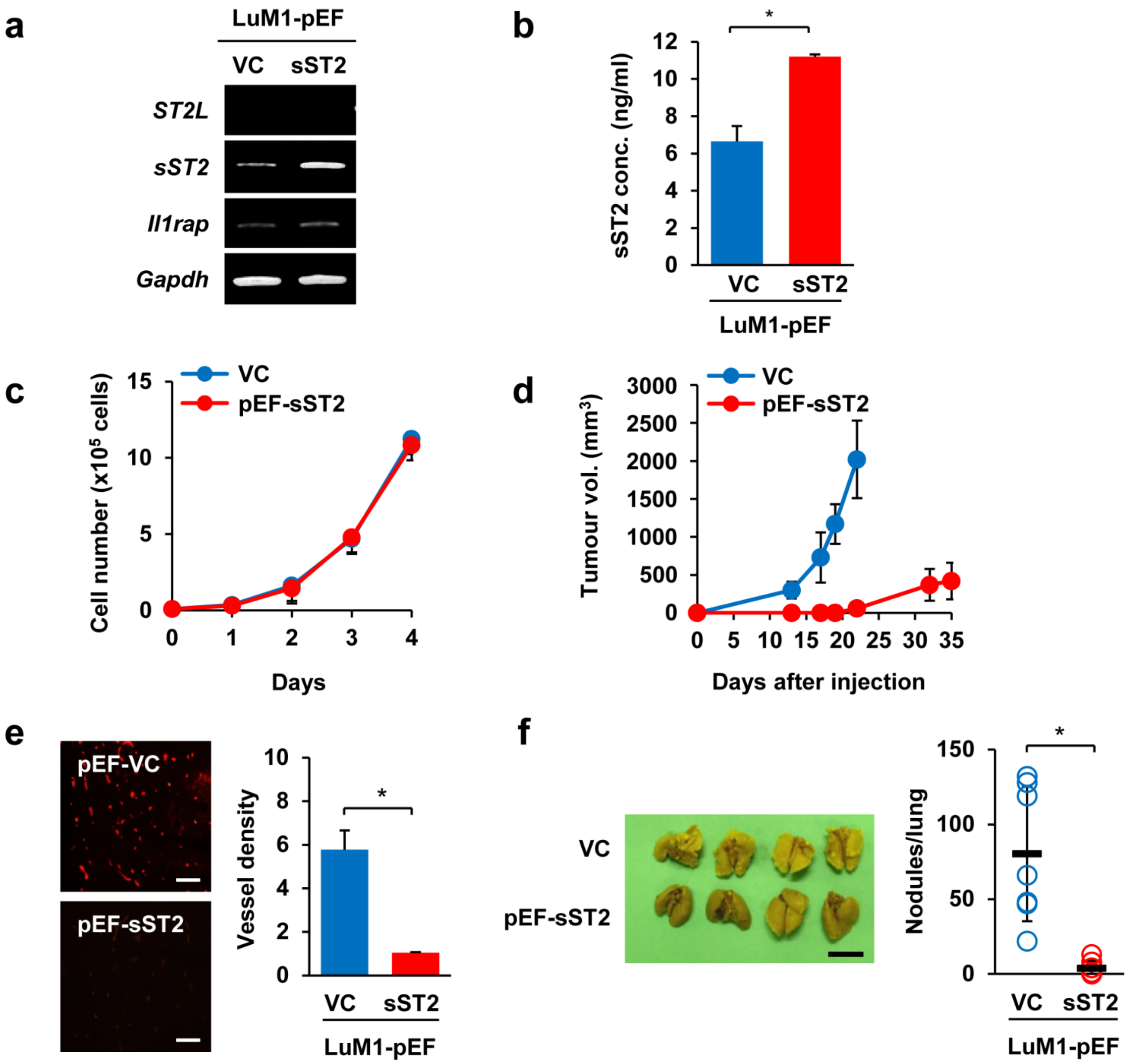

Supplementary Figure 2. Overexpression of sST2 inhibits the malignant growth of LuM1 cells. (**a**) Expression of S*T2L*, sST2 and *Il1rap* mRNA in LuM1 cells transfected with the pEF plasmid (LuM1-pEF-VC) or with pEF plasmid expressing mouse sST2 (LuM1-pEF-sST2). *Gapdh* served as the loading control. (**b**) Secretion of sST2 by LuM1-pEF-VC and LuM1-pEF-sST2 cells (n=3). (**c**) *In vitro* growth of LuM1-pEF-VC and LuM1-pEF-sST2 cells (n=3). (**d**) Growth of LuM1-pEF-VC and LuM1-pEF-sST2 tumours in BALB/c mice (n=7 mice per group). (**e**) Vessel density in LuM1-pEF-VC (n=15 fields) and LuM1-pEF-sST2 tumours. CD31 staining (left) and vessel density (right) (n=15 fields). Bar: 200 μm. (**f**) Spontaneous lung metastatic ability of LuM1-pEF-VC and LuM1-pEF-sST2 cells (n=7 mice per group). Bar: 1 cm. The data are shown as the mean ± s.d. * P<0.001 by Student's t-test.

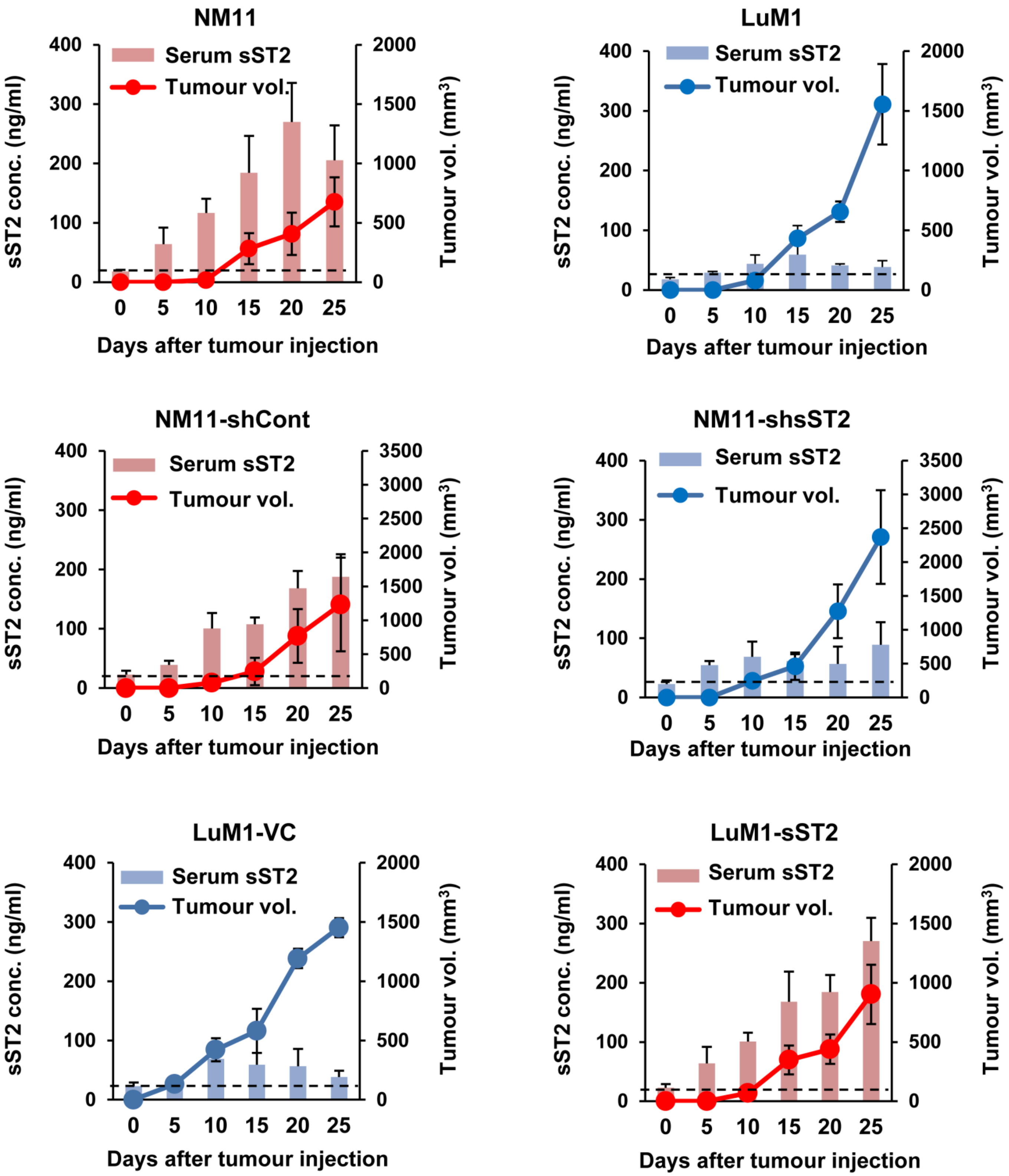

Supplementary Figure 3. sST2 serum concentrations in tumour-bearing mice. Cells (3×10^5 cells) were inoculated subcutaneously into 6-week-old female BALB/c mice (n=6 mice per group). The tumour sizes were measured and serum samples were collected every 5 days. sST2 serum concentrations were determined by ELISA. The dashed lines indicate the mean value of the sST2 serum concentration (17.9 ng/ml) in non-tumour-bearing age-matched BALB/c mice (n=5 mice per group). The data are shown as the mean ± s.d..

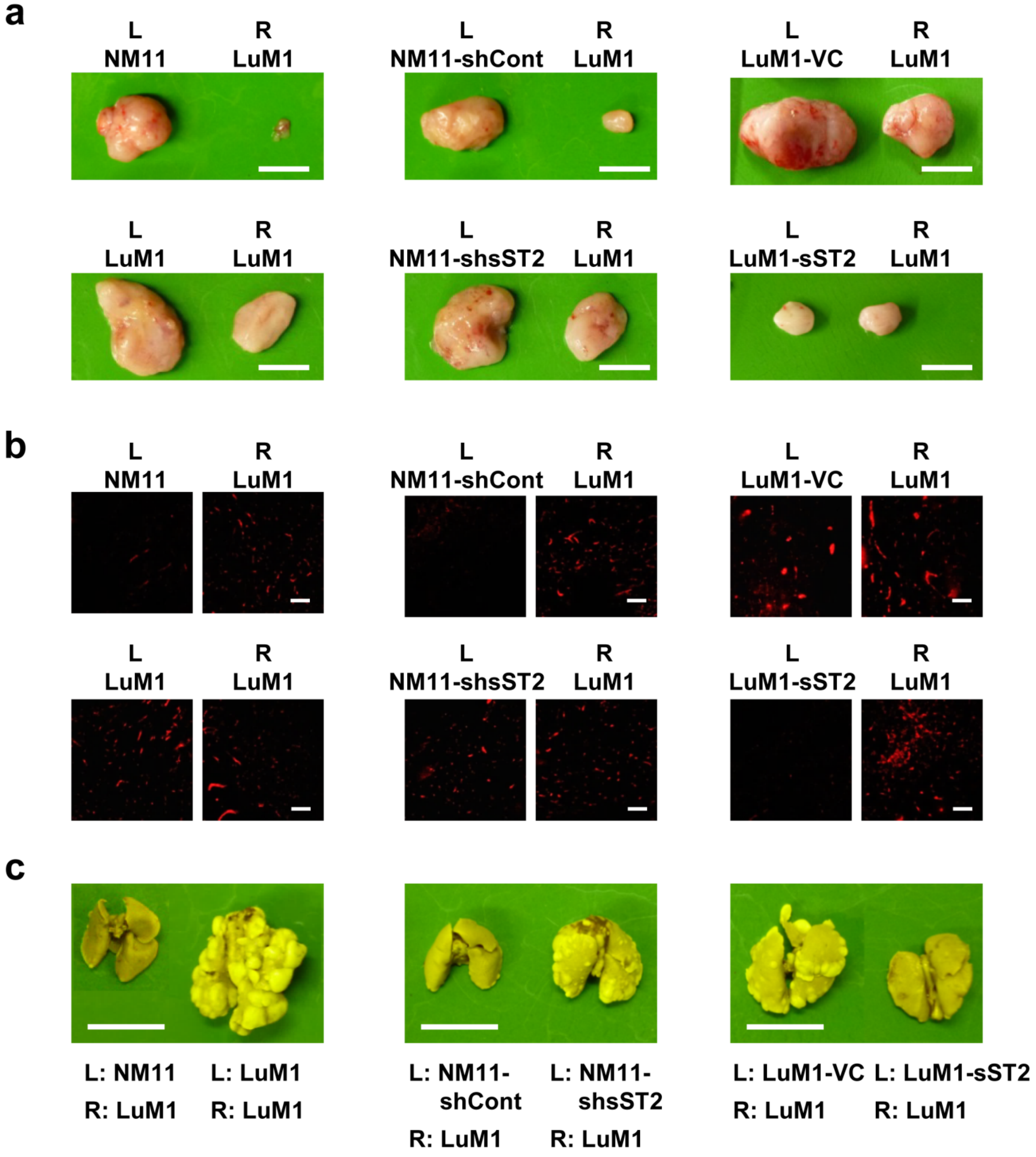

Supplementary Figure 4. Circulating sST2 suppresses the malignant growth of distant LuM1 tumours. BALB/c mice were subcutaneously inoculated with sST2 high-expressing (NM11-shCont and LuM1-sST2) or low-expressing cells (LuM1-VC and NM11-shsST2) into the left flank (L) on Day 0, followed by the injection of LuM1 cells into the right flank (R) on Day 5. The mice were euthanized on Day 30, and the tumours and lungs were collected. (**a**) Tumour growth. Bars, 1 cm. (**b**) Angiogenesis. CD31 staining of the cryostat sections of tumours is shown. Bars, 200 μm. (**c**) Lung metastases. Bars, 1 cm.

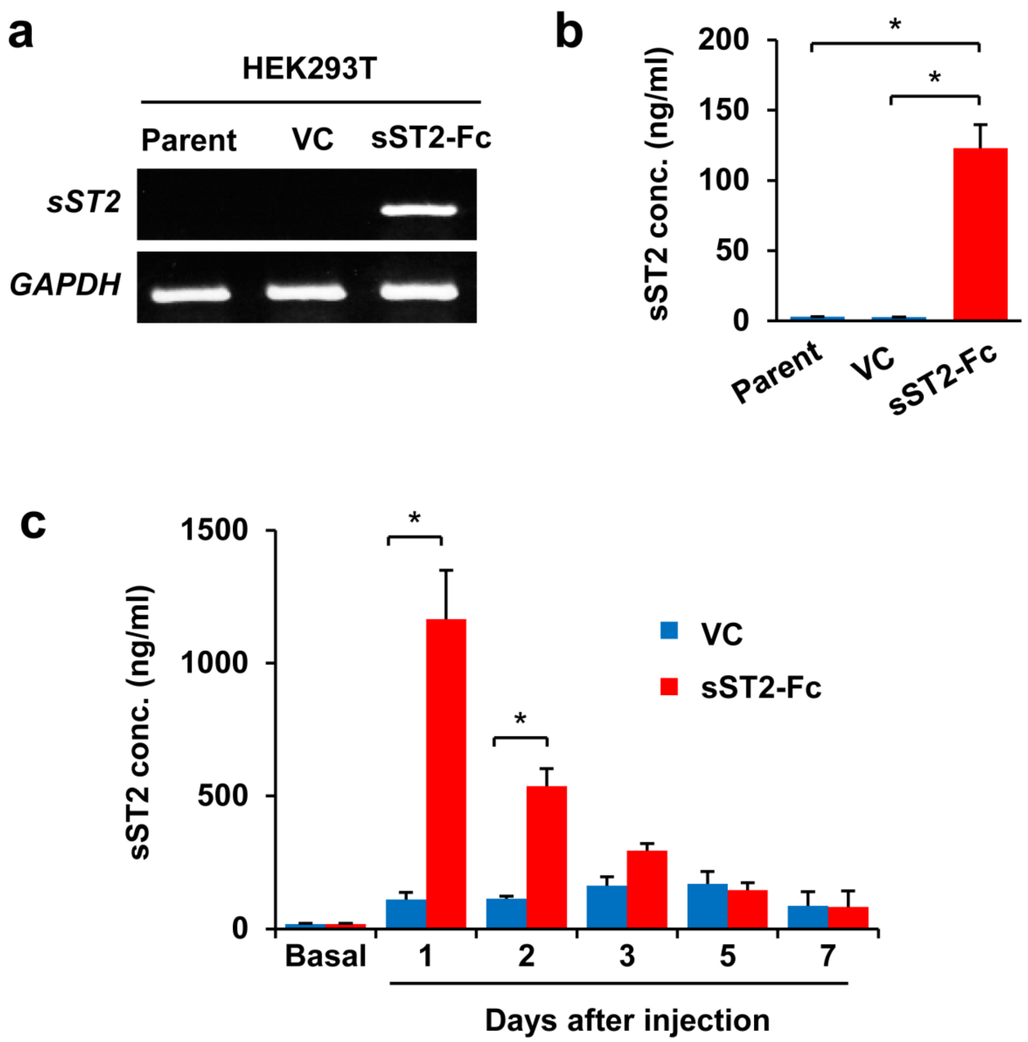

Supplementary Figure 5. Expression of the sST2-Fc fusion protein in HEK293T cells and BALB/c mice hydrodynamically transfected with the expression vector. (**a**) RT-PCR analysis of *sST2-Fc* mRNA in parental HEK293T cells, cells transfected with pcDNA3.1 (VC) and cells transfected with the pcDNA3.1/sST2-Fc expression vector (sST2-Fc). (**b**) Secretion of the sST2-Fc fusion proteins into culture supernatants by parental HEK293T cells, cells transfected with pcDNA3.1 (VC) and cells transfected with the pcDNA3.1/sST2-Fc expression vector (sST2-Fc). The conditioned media were collected 2 days after transfection, and the concentrations of the sST2-Fc fusion proteins were measured (n=3). (**c**) Production of sST2-Fc fusion proteins in BALB/c mice hydrodynamically injected with pcDNA3.1 alone (VC) and the pcDNA3.1/sST2-Fc expression vector (sST2-Fc). On Days 1, 2 3, 5 and 7 after injection (n=5 mice per group), sST2 serum concentrations were measured by ELISA. The data are shown as the mean ± s.d.. * $P<0.01$ by Student's *t*-test.

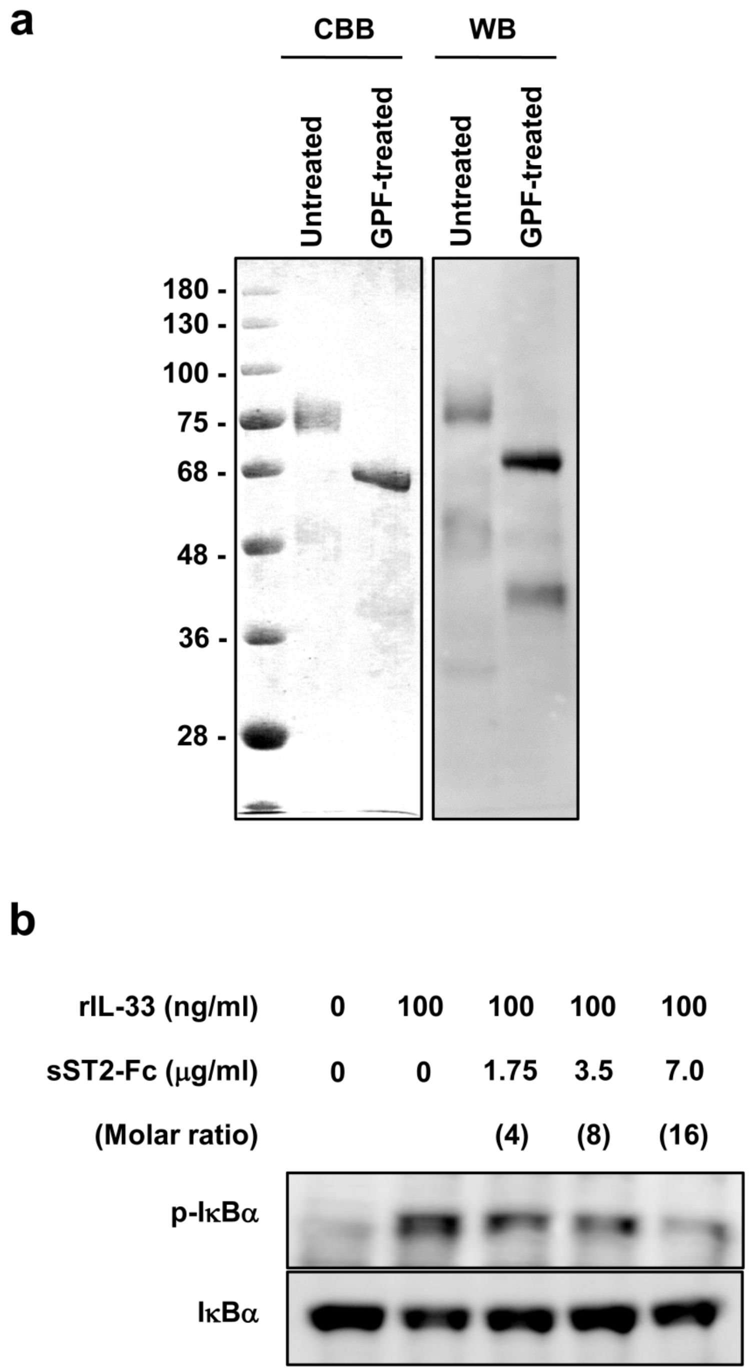

Supplementary Figure 6. Preparation of sST2-Fc fusion protein. (**a**) Preparation of sST2-Fc fusion protein. sST2-Fc protein released into the medium by ExpiCHO-S cells transfected with the sST2-Fc expression plasmid (pcDNA3.1-sST2-Fc) was isolated using an nProtein A Sepharose column. The purity of the sST2-Fc fusion protein was evaluated by SDS-PAGE followed by Coomassie brilliant blue (CBB) staining and Western blotting (WB) using anti-ST2 antibody. Glycosylation of the fusion protein was confirmed after treatment with glycopeptidase F (GPF). (**b**) Biological activity of the sST2-Fc protein. Biological activity was evaluated by the suppression of IκBα phosphorylation induced by mouse IL-33 (100 ng/ml) in P29 cells.

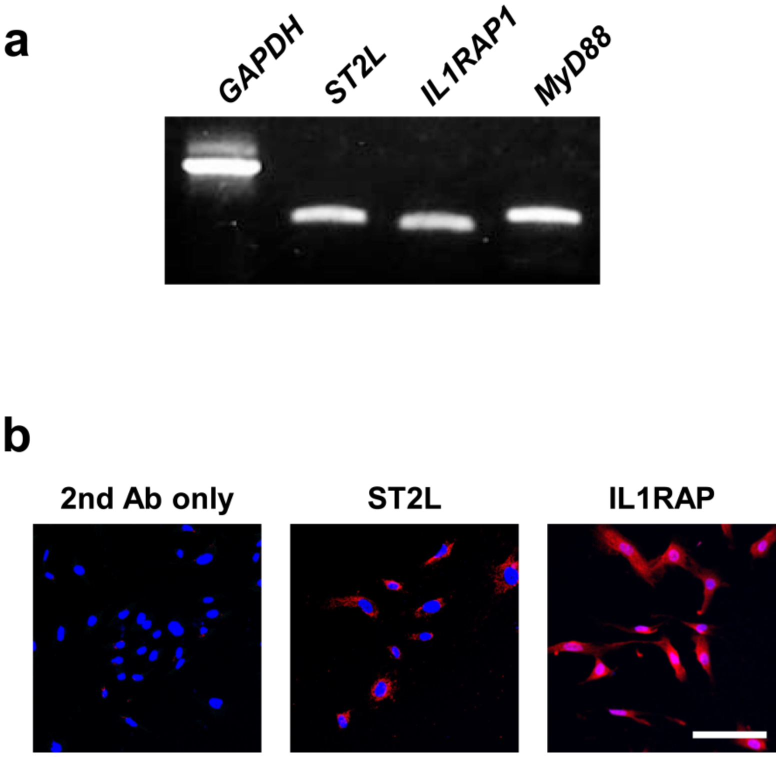

Supplementary Figure 7. Expression of ST2L, IL-1RAP and MyD88 in HUVECs. (**a**) RT-PCR analysis of *ST2L*, *IL1RAP* and *MyD88* mRNA expression. (**b**) Immunofluorescent staining of ST2L and IL-1RAP. Bar: 50 μm.

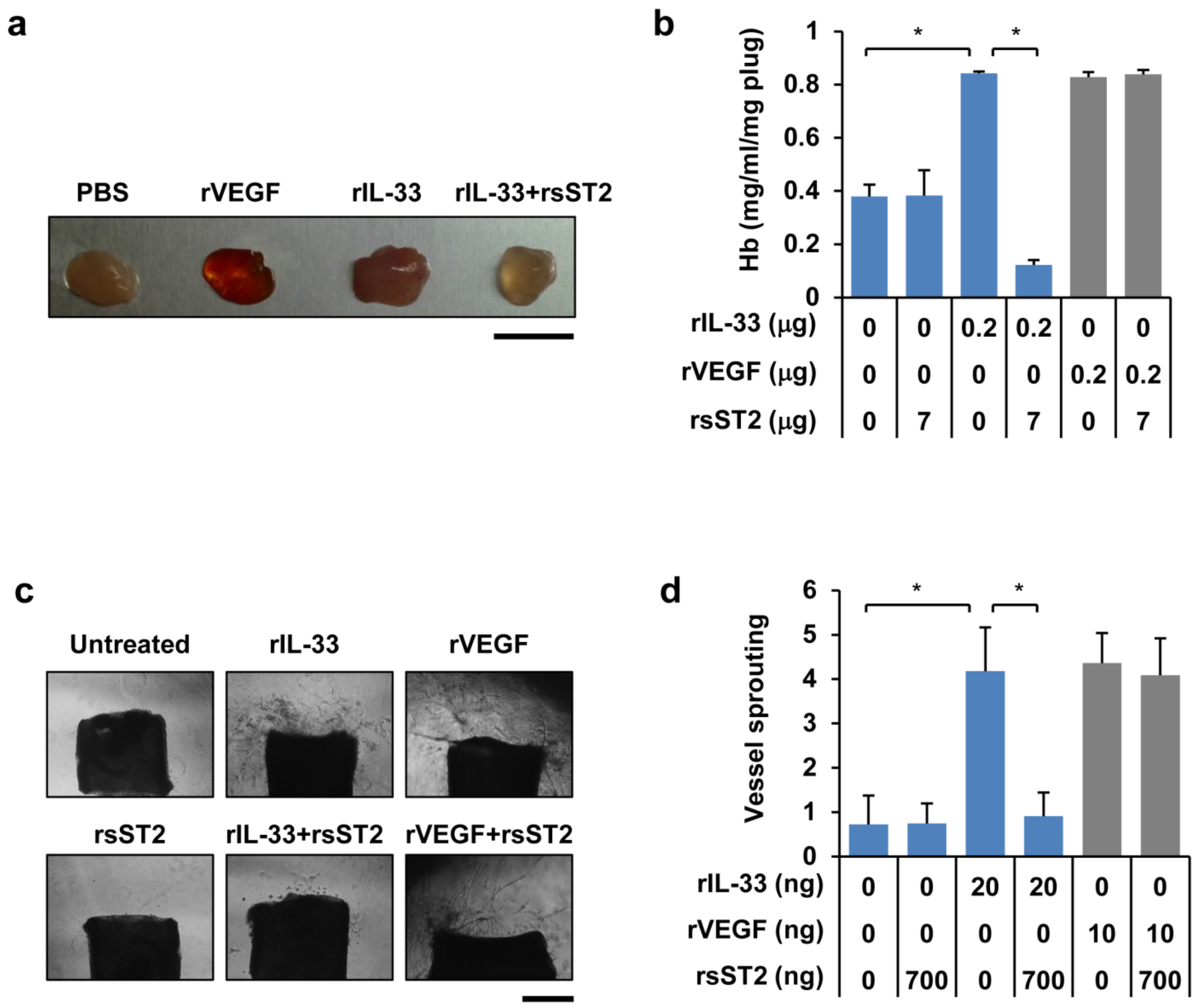

Supplementary Figure 8. Effect of sST2 on IL-33-induced angiogenesis in the Matrigel plug assay and the aortic ring assay. (**a**, **b**) Matrigel plug assay. Six-week-old BALB/c mice (n=3 mice per group) were injected subcutaneously with 0.5 ml Matrigel containing the indicated amount of rIL-33 with or without rsST2. After 6 days, the plugs were homogenized in PBS, and the total haemoglobin concentrations were measured by the Drabkin method. Bar: 1 cm. (**c**, **d**) Aortic ring sprouting assay. Aortic rings on Matrigel were incubated with 20 ng/ml rIL-33 protein with or without 700 ng/ml rsST2-Fc for 2 weeks. The angiogenic sprouting from aortic rings (n=4) was photographed, and the sprouting was quantified by counting the number of vascular sprouts that directly originated from the aorta. Bar: 500 μm. The data are shown as the mean ± s.d.. *P<0.001 by Student's t-test.

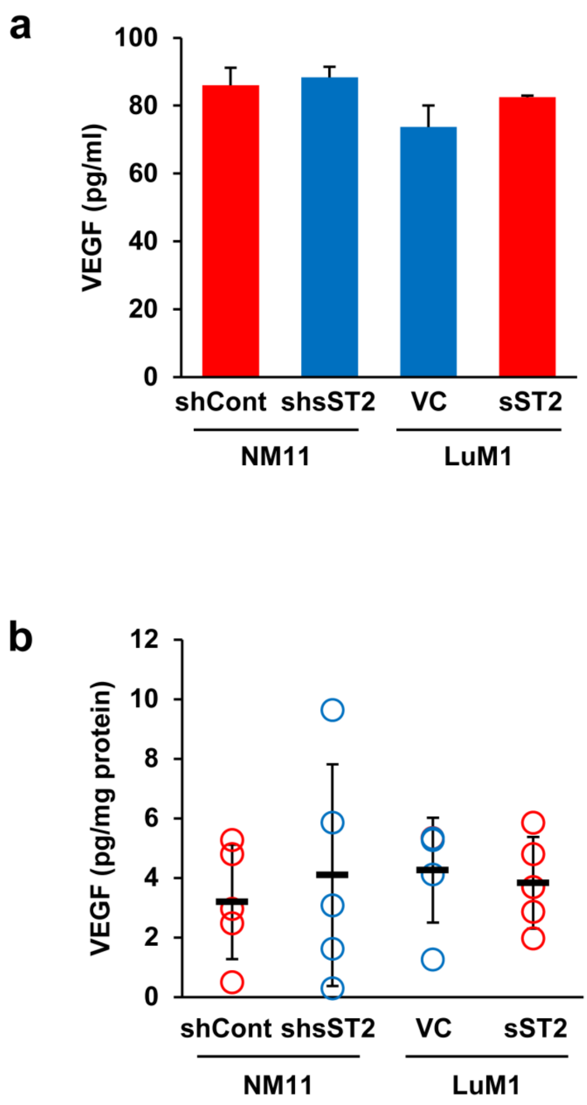

Supplementary Figure 9. VEGF production by cells and VEGF concentrations in the tumours. (**a**) VEGF production by NM11-shCont, NM11-shsST2, LuM1-VC and LuM1-sST2 cells. The cells were cultured for 2 days, and VEGF concentrations in the conditioned media were measured by ELISA (n=3). (**b**) VEGF concentrations in the tumours. VEGF concentrations in the tumour lysates established by NM11-shCont, NM11-shsST2, LuM1-VC and LuM1-sST2 cells were measured by ELISA (n=5). The data are shown as the mean ± s.d..

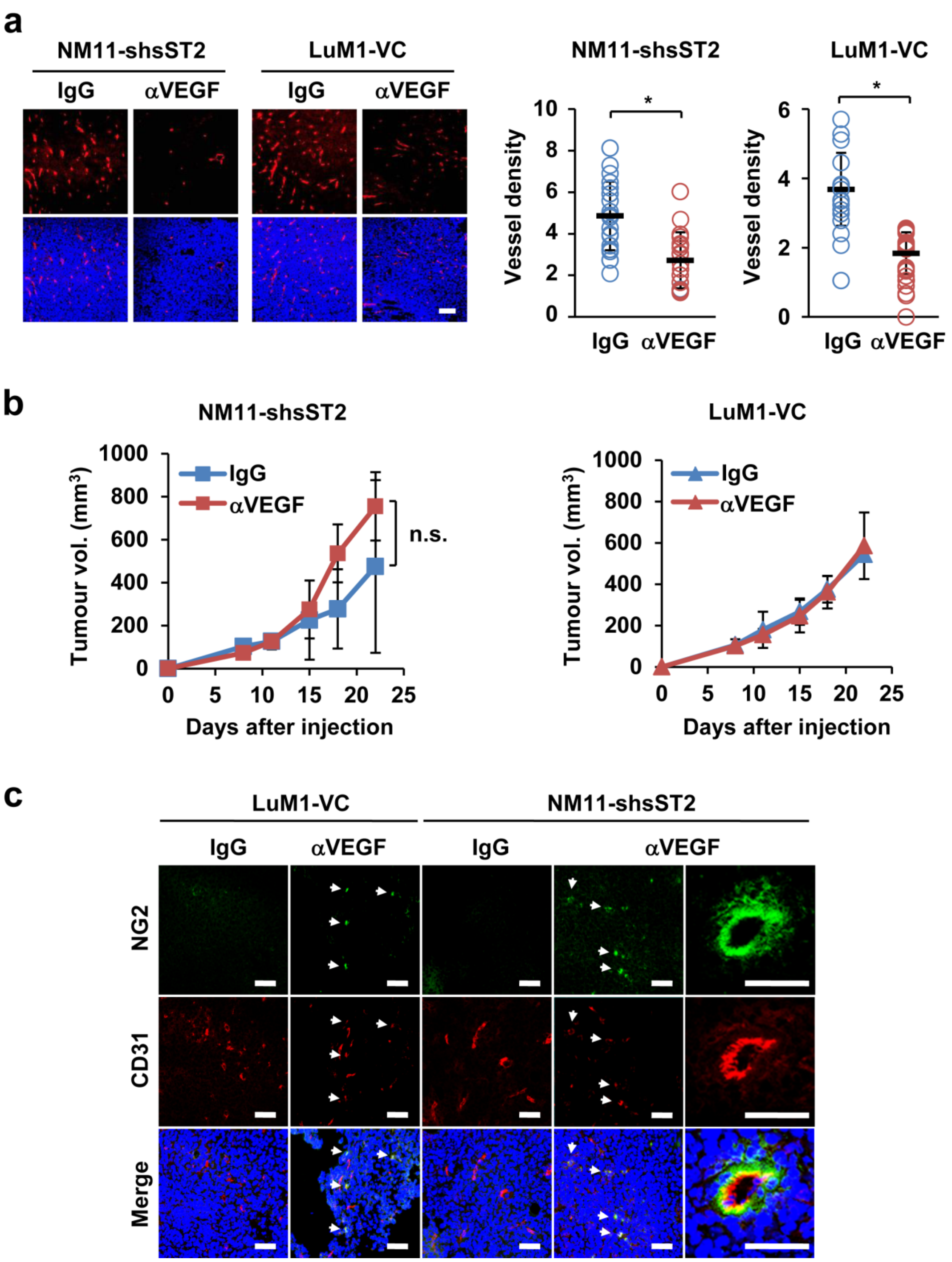

Supplementary Figure 10. Effect of anti-VEGF treatment on the angiogenesis and tumour growth of CRC cells. Balb/c female mice were subcutaneously injected with NM11-shsST2 and LuM1-VC cells (n=4 per group). The mice were intraperitoneally administered twice a week (total 7 times) with isotype-matched IgG (100 μg/mouse) or monoclonal anti-VEGF antibody (100 μg/mouse) starting on day 1 post-injection of tumour cells. (**a**) Tumour angiogenesis. Left: CD31 staining. Right: vessel density. NM11-shsST2 tumours (n=29 fields), LuM1-VC tumours (n=30 fields). Bar: 100 μm. (**b**) Tumour growth. (**c**) Pericyte coverage of microvessels. Cryosections of the tumours were immunostained with anti-CD31 and anti-NG2 antibodies. The sections were counterstained with DAPI. The arrows indicate vessels with pericytes. Bars, 100 μm. The data are shown as the mean ± s.d.. n.s., not significant. *P<0.001 by Student's t-test.

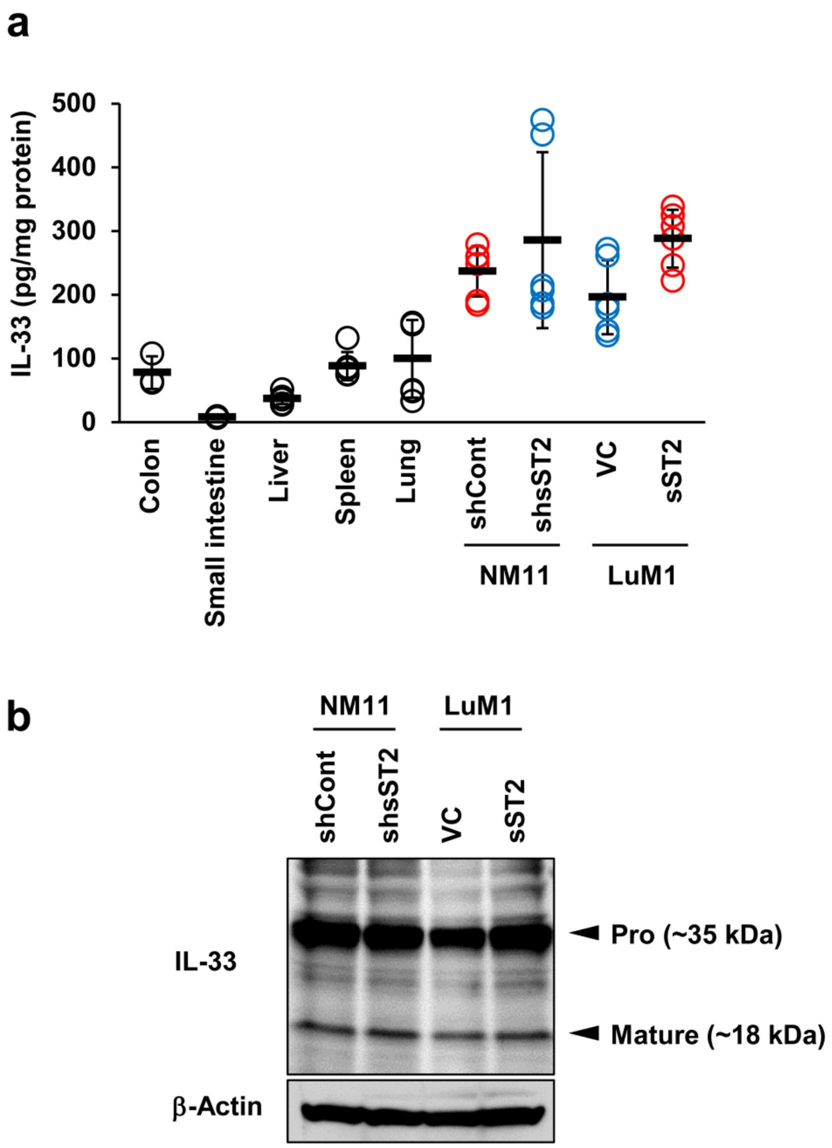

Supplementary Figure 11. IL-33 concentrations and processing in tumour tissues. (**a**) IL-33 concentrations in the tumours. IL-33 concentrations in the lysates of normal tissues (colon, small intestine, liver, spleen and lung) (n=5) and in tumour lysates (n=5) established by NM11-shCont, NM11-shsST2, LuM1-VC and LuM1-sST2 cells were measured by ELISA. (**b**) Processing of IL-33 in the tumours. IL-33 in the lysates of NM11-shCont, NM11-shsST2, LuM1-VC and LuM1-sST2 tumours was detected by SDS-PAGE followed by Western blotting with an anti-IL-33 antibody. β-Actin served as the loading control.

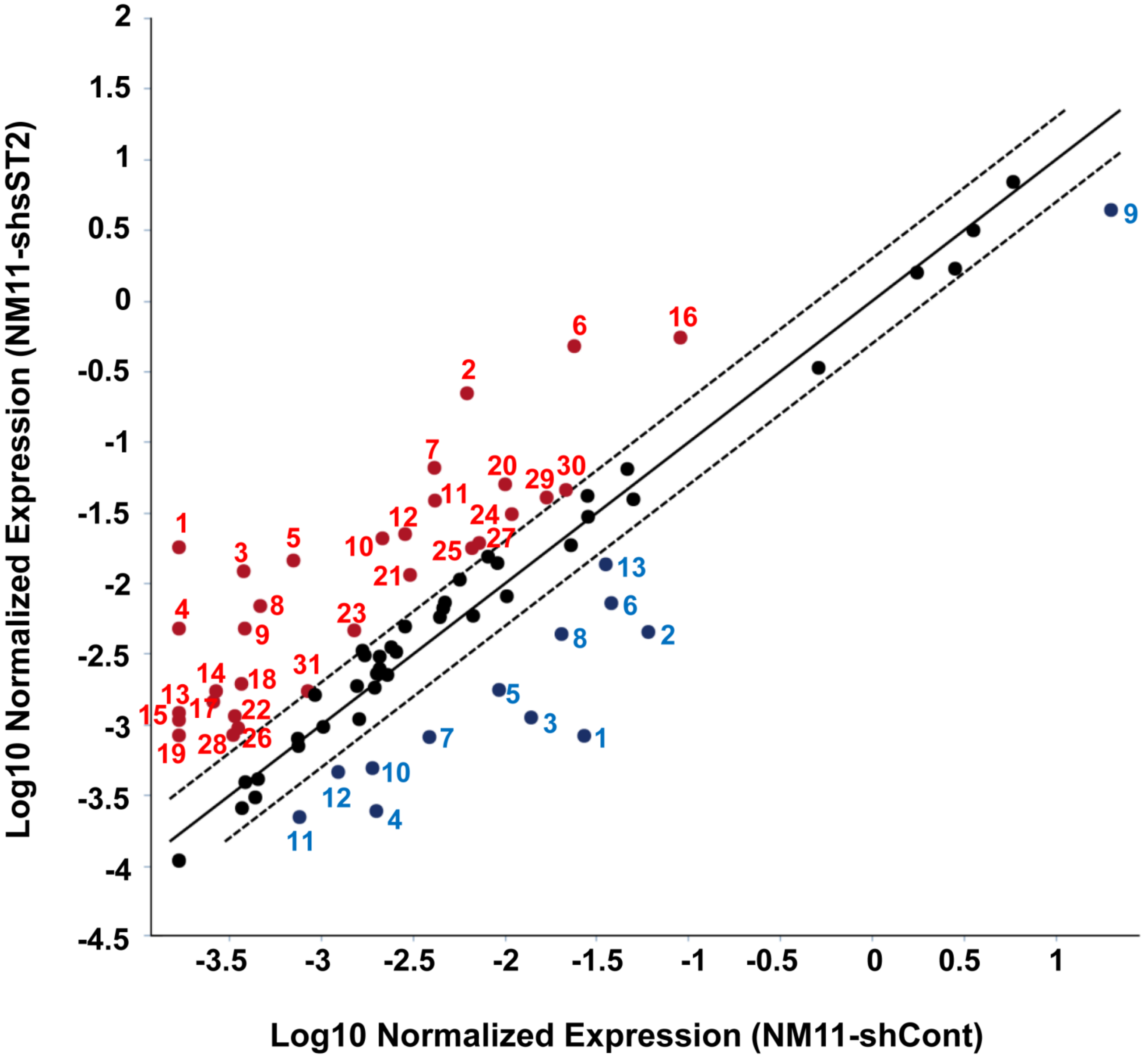

Supplementary Figure 12. Differential gene expression of chemokines and cytokines in sST2-knockdown NM11 cells. Profiling of cytokine and chemokine genes. Chemokine and cytokine genes differentially expressed in NM11-shsST2 versus NM11-shCont cells were profiled by a Mouse Cytokines & Chemokines RT[2] Profiler PCR Array. The numbers correspond to those in Supplementary Table 1.

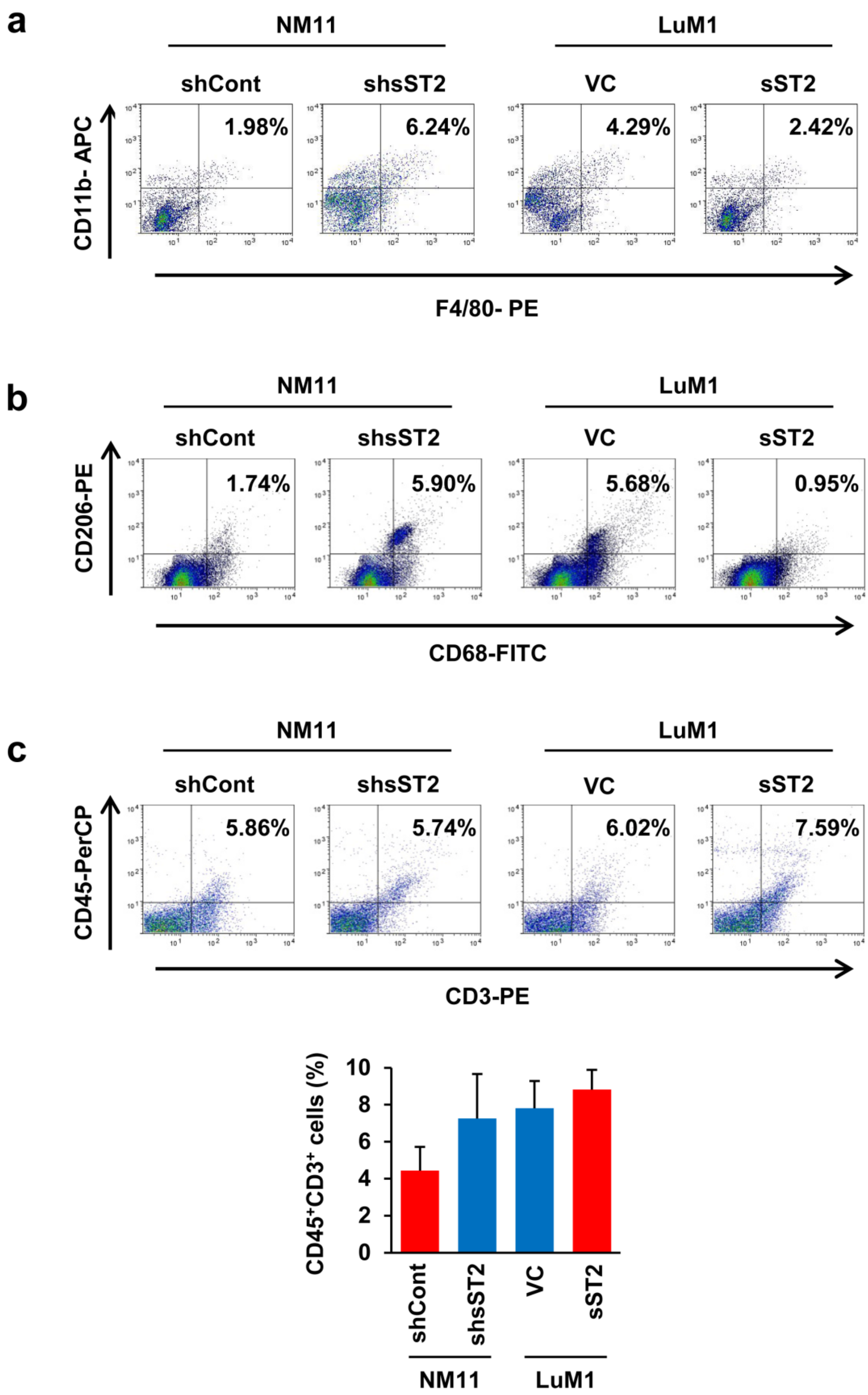

Supplementary Figure 13. FACS analysis of macrophage infiltration and polarization and CD45$^+$CD3$^+$ lymphoid cells in tumours. (**a**) Macrophage infiltration. Cells in tumours established by NM11-shCont, NM11-shsST2, LuM1-VC and LuM1-sST2 cells were immunostained with anti-F4/80-PE and anti-CD11b-APC and analysed by FACS. (**b**) Macrophage polarization of the M2a subset. Macrophages in the tumours (CD68$^+$CD206$^+$) were analysed by FACS. (**c**) CD45$^+$CD3$^+$ lymphoid cells in the tumours. CD45$^+$CD3$^+$ lymphoid cells were analysed by FACS (n=3).

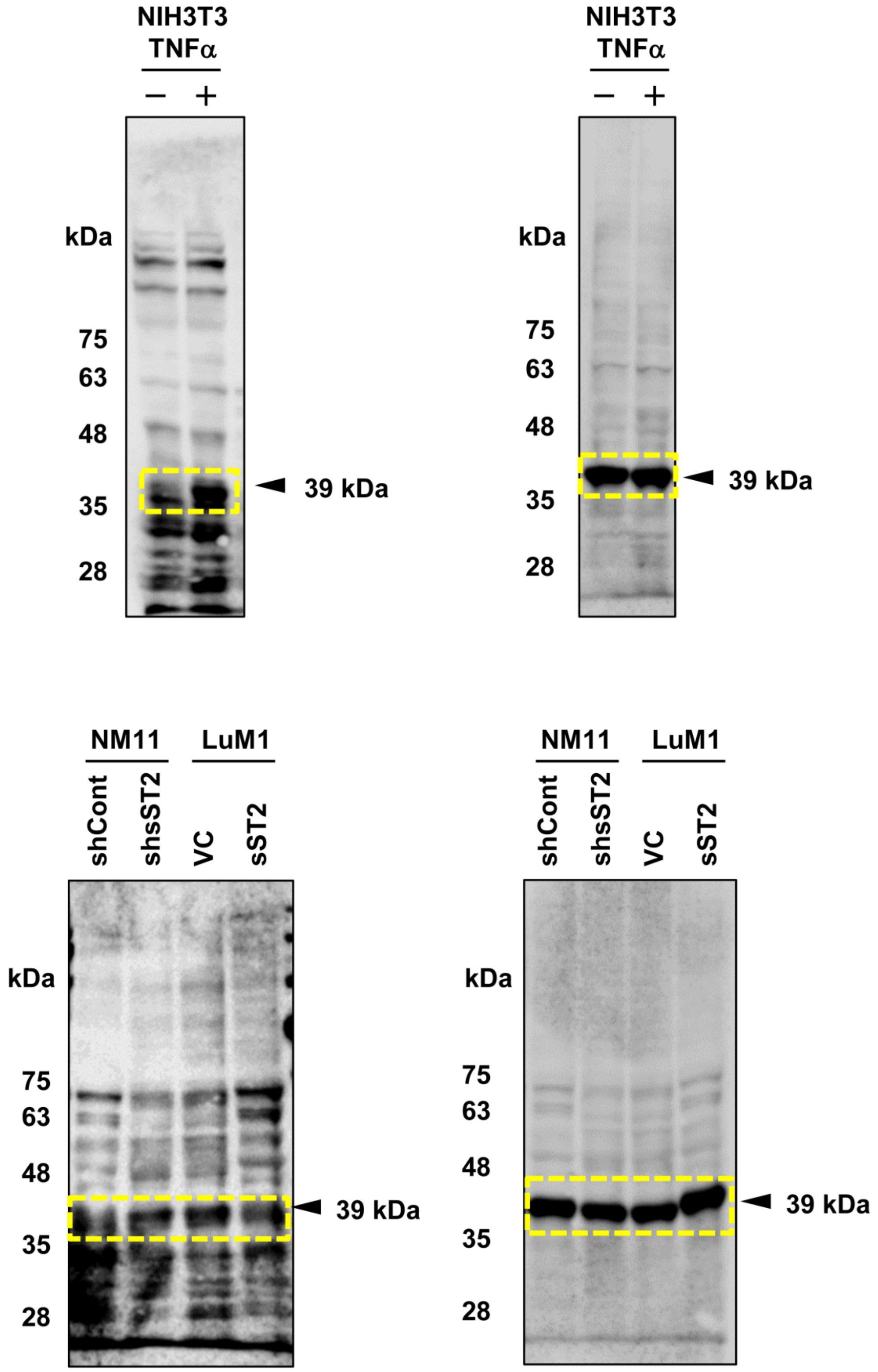

Supplementary Figure 14. Full-size images of Western blots. The yellow dotted line indicates the cropped region included in Figure 6a.

Supplementary Table 1. Differential gene expression of chemokines and cytokines in sST2-knockdown NM11 cells.

Up-regulated

No.	Symbol	NM11-shsST2	NM11-shCont	shsST2/shCont
1	*Il17f**	0.017886	0.000166	107.75
2	*Ccl7*	0.219912	0.006162	35.69
3	*Il13*	0.012132	0.000375	32.35
4	*Il4*	0.004759	0.000166	28.67
5	*Cxcl3*	0.014428	0.000699	20.64
6	*Ccl12*	0.474671	0.023642	20.08
7	*Cxcl10*	0.06538	0.004093	15.97
8	*Cxcl9*	0.006872	0.000461	14.91
9	*Ppbp*	0.004759	0.00038	12.52
10	*Tnfsf11*	0.020689	0.002134	9.70
11	*Il1rn**	0.03834	0.004122	9.30
12	*Cxcl5*	0.022174	0.002835	7.82
13	*Nodal*	0.001206	0.000166	7.27
14	*Tgfb2*	0.001718	0.000265	6.48
15	*Fasl*	0.001072	0.000166	6.46
16	*Ccl2*	0.545254	0.089467	6.10
17	*Bmp7*	0.001445	0.000256	5.64
18	*Il12b**	0.001933	0.000364	5.31
19	*Ifng**	0.000836	0.000166	5.04
20	*Csf1*	0.049894	0.00994	5.02
21	*Cxcl1*	0.011399	0.003017	3.78
22	*Cxcl11**	0.001141	0.000335	3.41
23	*Xcl1*	0.004629	0.001498	3.10
24	*Il1b**	0.030713	0.010802	2.84
25	*Ccl3*	0.00094	0.000349	2.70
26	*Ccl1*	0.01764	0.006558	2.69
27	*Il18*	0.01917	0.007177	2.67
28	*Il10**	0.000841	0.000328	2.56
29	*Ccl4*	0.040246	0.016717	2.41
30	*Ccl5**	0.045594	0.021307	2.14
31	*Tnf*	0.001718	0.000837	2.05

Down-regulated

No.	Symbol	NM11-shsST2	NM11-shCont	shsST2/shCont
1	*Tnfrsf11b*	0.00083	0.026784	0.03
2	*Cxcl13*	0.004503	0.059850	0.08
3	*Il7*	0.001118	0.013768	0.08
4	*Ccl20*	0.000243	0.001977	0.12
5	*Il11**	0.001754	0.009211	0.19
6	*Cxcl12*	0.007214	0.037616	0.19
7	*Ccl19**	0.000813	0.003846	0.21
8	*Bmp4*	0.004349	0.020158	0.22
9	*Spp1*	4.362031	19.800981	0.22
10	*Il16*	0.00049	0.001883	0.26
11	*Il12a**	0.000221	0.000754	0.29
12	*Lta*	0.00046	0.001225	0.38
13	*Ltb*	0.013555	0.035097	0.39

Upregulated and downregulated genes in NM11-shsST2 cells. Direct NF-κB target genes are marked with asterisks. See Supplementary Figure 12.

Supplementary Table 2. Antibodies used for IHC, IF and FCM analyses.

Antibodies	Clone	Label	Supplier Catalogue #	Application	Dilutions Conc.
LEAF™ Purified Rat IgG2a, κ Isotype Ctrl Antibody	RTK2758	-	BioLegend 400502	Neutralization (control antibody)	-
LEAF™ Purified anti-mouse VEGF-A Antibody	2G11-2A05	-	BioLegend 512808	Neutralization	-
Rabbit anti-human IL1RL1	-	-	Atlas Antibodies HPA007406	IHC	1:500
Mouse anti-human CD31	JC70A	-	DAKO IS610	IHC	1:100
Rabbit anti-NG2	-	-	Cell Signaling #4235	IF	1:100
Rat anti-mouse CD31	MEC 13.3	-	BD Biosciences 553370	IF	1:100
Rat anti-mouse F4/80	A3-1	-	Bio-Rad MCA497G	IF	1:100
Goat anti-mouse ST2/IL-1R4	-	-	R&D Systems AF1004	IF	1:200
Mouse anti-human ST2	HB12	-	MBL D065-3	IF	1:200
Rabbit anti-IL1RAP	-	-	Abcam Ab8110	IF	1:150
Rat anti-mouse CD16/32	93	-	BioLegend 101301	FCM	1 μg/2x10^6 cells
Rat anti-mouse CD45	30-F11	PerCP /Cy5.5	BD Biosciences 561869	FCM	1 μg/2x10^6 cells
Hamster anti-mouse CD3e	145-2C11	PE	BD Biosciences 561824	FCM	1 μg/2x10^6 cells
Rat anti-mouse F4/80	A3-1	PE	BioLegend 122615	FCM	1 μg/2x10^6 cells
Rat anti-mouse CD11b	M1/70	APC	BD Biosciences 561690	FCM	1 μg/2x10^6 cells
Rat anti-mouse CD68	FA-11	FITC	BIO-Rad MCA1957FT	FCM	1 μg/2x10^6 cells
Rat anti-mouse CD206	MR5D3	PE	BIO-Rad MCA2235PET	FCM	1μg/2x10^6 cells
Rat anti-mouse IL-10	JES5-16E3	PerCP /Cy5.5	BioLegend 505027	FCM	1 μg/2x10^6 cells
Rat anti-mouse IL-12/ IL-23p40	C15.6	APC	BioLegend 505205	FCM	1 μg/2x10^6 cells
Rat IgG1,κ Isotype Ctrl	RTK2071	APC	BioLegend 400411	FCM	1 μg/2x10^6 cells
Rat IgG2b,κ Isotype Ctrl	RTK4530	APC	BioLegend 400611	FCM	1 μg/2x10^6 cells
Rat IgG2b, κ Isotype Ctrl	RTK4530	PerCP /Cy5.5	BioLegend 400631	FCM	1 μg/2x10^6 cells
Armenian Hamster IgG Isotype Ctrl	HTK888	PE	BioLegend 400907	FCM	1 μg/2x10^6 cells
Rat IgG2b, κ Isotype Ctrl	RTK4530	PE	BioLegend 400607	FCM	1 μg/2x10^6 cells
Rat IgG2a, κ Isotype Ctrl	RTK2758	FITC	BioLegend 400505	FCM	1 μg/2x10^6 cells

IHC; immunohistochemistry, IF; immunofluorescence, FCM; flow cytometry

Supplementary Table 3. Primers used for PCR and real-time PCR.

Primer name	Sequences (5' to 3')	Accession No.	Application
moST2L-F	GCATGATAAGGCACACCATAA	NM_001025602	PCR, qPCR
moST2L-R	ATCGTAGAGCTTGCCATCGT		
mosST2-F	GCATGATAAGGCACACCATAA	NM_010743	PCR, qPCR
mosST2-R	ACACAGAGAGGGGAAGGATA		
moIl1rap-F	TCCTCTGGCCTTACCCTGATCT	NM_134103	PCR
moIl1rap-R	AACCCTTATACCAAGTGACCG		
moMyd88-F	GTGGTGGTTGTTTCTGACGA	NM_010851	PCR
moMyd88-R	AGGGTCATCTTCAGGGCAG		
moIl33-F1	GGAAGAAGGTGATGGTGAAC	NM_133775	PCR
moIl33-R1	CCACAACATCGTAAGCCAAG		
moIl33-F2	GGAAAAGACCAAGAGCAAGACC	NM_001164724	qPCR
moIl33-R2	TTCTTCCCATCCACACCGTC		
huST2L-F	GAAGGCACACCGTAAGACTA	NM_016232	PCR, qPCR
huST2L-R	TTGTAGTTCCGTGGGTAGAC		
husST2-F	GAAGGCACACCGTAAGACTA	NM_003856	PCR, qPCR
husST2-R	GACAAACCAACGATAGGAGG		
huIL1RAP-F	CACTTCTGTGGTGTGTAGTGA	NM_002182	PCR
huIL1RAP-R	AATGCAACTTTGCTG CAATAT		
huMYD88-F	AAAGAGGTTGGCTAGAAGGC	NM_001172567	PCR
huMYD88-R	CAAGGCGAGTCCAGAACCA		
huIL33-F1	GTGACGGTGTTGATGGTAAG	NM_033439	PCR
huIL33-R1	CCAAGACTCACAGGTTTCCA		
huIL33-F2	CTGCCTGTCAACAGCAGTCT	NM_001164724	qPCR
huIL33-R2	CTGGTCTGGCAGTGGTTTTT		
GAPDH-F	ACCACAGTCCATGCCATCAC	NM_001256799 (hu)<br>NM_001289726 (mo)	PCR
GAPDH-R	TCCACCACCCTGTTGCTGTA		
moGapdh-F	TGCACCACCAACRGCTTAG	NM_008084	qPCR
moGapdh-R	GGATGCAGGGATGATGTTC		
huGAPDH-F	CGCTCTCTGCTCCTCCTGTT	NM_002046	qPCR
huGAPDH-R	CCATGGTGTCTGAGCGATGT		
moIfng-F	GGCCATCAGCAACAACATAAGCGT	NM_008337	qPCR
moIfng-R	TGGGTTGTTGACCTCAAACTTGGC		
moIl4-F	AGATGGATGTGCCAAACGTCCTCA	NM_021283	qPCR
moIl4-R	AATATGCGAAGCACCTTGGAAGCC		
moIl13-F	ACAAGACCAGACTCCCCTGT	NM_008355	qPCR
moIl13-R	TCTGGGTCCTGTAGATGGCA		
moCcl7-F	GATCTCTGCCACGCTTCTGT	NM_013654	qPCR
moCcl7-R	ATAGCCTCCTCGACCCACTT		
Alu-F	CACCTGTAATCCCAGCACTTT	-	*Alu* analysis
Alu-R	CCCAGGCTGGAGTGCAGT		
moGapdh-F	GCACAGTCAAGGCCGAGAAT	-	
moGapdh-R	GCCTTCTCCATGGTGGTGAA		

hu: human, mo: mouse
